# Abundant kif21b is associated with accelerated progression in neurodegenerative diseases

**DOI:** 10.1186/s40478-014-0144-4

**Published:** 2014-10-03

**Authors:** Karim L Kreft, Marjan van Meurs, Annet F Wierenga-Wolf, Marie-Jose Melief, Miriam E van Strien, Elly M Hol, Ben A Oostra, Jon D Laman, Rogier Q Hintzen

**Affiliations:** 1 Department Neurology Erasmus MC, University Medical Center, Room Ba 4.92, Rotterdam, 3000 CA The Netherlands; 2 Department Immunology Erasmus MC, University Medical Center, Rotterdam, The Netherlands; 3 Department MS Center ErasMS Erasmus MC, University Medical Center, Rotterdam, The Netherlands; 4 Department Clinical Genetics Erasmus MC, University Medical Center, Rotterdam, The Netherlands; 5 Astrocyte Biology & Neurodegeneration, Netherlands Institute for Neuroscience, An Institute of the Royal Academy of Arts and Sciences, Amsterdam, The Netherlands; 6 Swammerdam Institute for Life Sciences, Center for Neuroscience, University of Amsterdam, Amsterdam, The Netherlands; 7 Department Translational Neuroscience, University Medical Center Utrecht, Utrecht, The Netherlands

**Keywords:** Brain cortex, Astrocytes, Glioma, Kinesins, EDSS

## Abstract

Kinesin family member 21b (kif21b) is one of the few multiple sclerosis (MS) risk genes with a presumed central nervous system function. Kif21b belongs to the kinesin family, proteins involved in intracellular transport of proteins and organelles. We hypothesised that kif21b is involved in the neurodegenerative component of MS and Alzheimer's (AD) disease. Post-mortem kinesin expression was assessed in 50 MS, 58 age and gender matched non-demented controls (NDC) and 50 AD. Kif21b expression was five-fold increased in AD compared to MS and NDC aged below 62 years (p = 8*10^-5^), three-fold between 62-72 years (p = 0.005) and not different above 72 years. No significant differences were observed between MS and NDC. In AD, kif21b expression was two-fold increased in Braak stage 6 (scoring for density of neurofibrillary tangles) compared with stage 5 (p = 0.003). In MS patients, kif21b correlated with the extent of grey matter demyelination (Spearman's rho = 0.31, p = 0.03). Abundant kif21b, defined as expression above the median, was associated with a two-fold accelerated development of the Kurtzke Expanded Disability Status Scale (EDSS) 6.0 (median time in low kif21b group 16 years vs. high kif21b 7.5 years, log-rank test p = 0.04) in MS. Given the genetic association of kif21b with MS, the results were stratified according to rs12122721[A] single nucleotide polymorphism (SNP). No association was found between kif21b expression or the time to EDSS 6 in kif21b risk SNP carriers compared to non-risk carriers. Kif21b was expressed in astrocytes in addition to neurons. Upon astrocyte activation, kif21b increased nine-fold. Abundant kif21b expression is associated with severe MS and AD pathology and with accelerated neurodegeneration independent of the kif21b risk SNP.

**Electronic supplementary material:**

The online version of this article (doi:10.1186/s40478-014-0144-4) contains supplementary material, which is available to authorized users.

## Introduction

Multiple sclerosis (MS) has classically been considered as an auto-immune disease of the white matter. However, the grey matter component of the pathology receives increasing attention, especially because axonal and neuronal degeneration occur already early in the disease [1]. This area of research received an impulse by novel MRI techniques to visualize grey matter lesions [2].

Recently, a large genome wide association study (GWAS) in MS patients identified 57 single nucleotide polymorphisms (SNP) associated with an increased risk to develop MS. Most of the (SNP) have important functions in immune cells, mainly in T-cell biology [3]. However, a few of these SNP may be involved in neurodegeneration. Kif21b is one of these SNP, which was also associated with MS risk in earlier studies [4].

Kif21b is a relatively poorly studied member of the kinesin family, expressed most profoundly in the CNS, especially in the dendrites of neurons [5]. The kinesin superfamily contains 45 genes, known as kinesins or kifs, which are divided into 15 different families. These kifs are mainly expressed in neurons, and are localised in axons and dendrites in the CNS. Members of the kinesin family consist of a motor domain, a binding site for microtubules, and a specific cargo binding domain for the transport of different molecules and organelles. Neuronal functioning and survival largely depend on intracellular microtubule mediated transport, which is especially well developed in neurons, because long distances need to be covered along the axon [6]. The exact function and cargo transported by kif21b are currently unknown. Genetic variation in some of the kinesin family members has been linked to neurodegenerative diseases like amyotrophic lateral sclerosis (ALS), Huntington's and Alzheimer's disease [7].

Dementia is characterized by memory loss, cognitive impairments and decline in intellectual performance. The majority of patients with dementia suffer from Alzheimer's disease (AD). Neuropathological changes include the extracellular deposition of Aβ peptide in plaques, reactive gliosis [8] and intraneuronal aggregation of hyperphosphorylated Tau in tangles. Tau is a microtubule associated protein and binds to tubulin [9], along which kinesins transport their cargo. In AD, Tau is abnormally phosphorylated and therefore dissociates from microtubuli [10], leading to the formation of neurofibrillary tangles (NFT). The level of NFT strongly correlates with neuronal dysfunction and clinical progression in AD [11]. The extend of Tau pathology is reflected in the Braak stage [12].

We hypothesized that kif21b is aberrantly expressed in neurodegenerative disease and that its expression is associated with disease progression. Therefore, kif21b expression was assessed in 50 MS patients compared with 58 non-demented controls (NDC) and with 50 Alzheimer's patients (AD). We found that cortical kif21b expression is significantly increased in AD patients compared with MS patients and NDC, independently of the MS risk genotype. Moreover, in AD and MS patients with more severe neuropathology, significantly higher expression levels of kif21b were found. This increased kif21b expression was associated with a shorter disease duration in both MS and AD patients and accelerated progression to sustained neurological disability (EDSS 6.0) in MS.

## Materials and methods

### Brain tissue

Brain and spinal cord tissue was obtained from the Netherlands Brain Bank (NBB), Netherlands Institute for Neuroscience, Amsterdam. The cortical tissue consisted of the medial temporal gyrus of 50 Alzheimer patients (including two AD patients: superior frontal gyrus tissue), 50 multiple sclerosis patients (including six tissues of MS patients from the superior frontal gyrus and one hippocampus) and 58 age and gender matched non-demented controls (all medial temporal gyrus). The majority of non-demented controls were free of neurological disease. However, three patients had cerebral or cerebellar micrometastases of carcinomas, one NDC had a small Schwannoma of the right vestibulocochlear nerve four NDC had old infarctions and one had mononeuropathy multiplex. None of the tumours or infarctions were located in the tissues sections studied here. White matter tissue was obtained from 18 NDC, 23 MS and three AD patients. Additionally, seven MS spinal cord samples were tested. The medical ethical committee of the Free University Medical Center Amsterdam, The Netherlands, has approved this study and all materials have been collected from donors from whom written informed consent for brain autopsy and the use of the material and clinical information for research purposes had been obtained by the NBB. Brain tissue was stored at -80°C until use.

### RNA isolation and real-time quantitative PCR

For isolation of total RNA 5-7 cryo-sections of 50 μm were used. RNA isolation was performed by using the GenElute Mammalian Total RNA Miniprep Kit (Sigma). RNA samples were treated with DNAse I to remove contaminating DNA (Invitrogen). Using 1.0 μg RNA as a template, copy DNA (cDNA) was reverse transcribed by using Superscript II (Invitrogen). Primers and probes were selected by using the Universal ProbeLibrary Assay Design Centre (Roche). To determine target gene mRNA expression, real-time quantitative reverse transcription PCR was performed using TaqMan technology. GAPDH mRNA and GUSB (for brain tissue) or 18S (for astrocyte cultures) RNA levels were measured as a control to normalize for RNA input. Both GUSB and 18S validated the GAPDH results. Reference gene primers and probes were obtained from Applied Biosystems. An Applied Biosystems 7900 Sequence Detector was programmed for the initial step of 2 min at 50°C and 10 min at 95°C, followed by 40 thermal cycles of 15 s at 95°C and 1 min at 60°C. For calculation of mRNA expression levels, Ct values per gene were applied to standard curves, generated for each gene of interest. Sequences of the primers are listed in Additional file 1: Table S1a.

### DNA isolation from brain tissue

5-10 mg of brain tissue was lysed using cell lysis solution (Qiagen) and Proteinase K (MP Biomedicals LLC) was added and incubated at 55°C over night. Samples were treated with RNAse A solution (Qiagen) and incubated at 37°C for 1 h. Protein precipitation solution (Qiagen) was added, and the samples were placed on ice for 5 min. 100% isopropanol was added to the supernatant and DNA was precipitated with 70% ethanol and hydrated in Tris EDTA buffer (TE-buffer) at 55°C for 5 min and stored at 4°C for SNP detection.

### SNP detection in brain tissue

Risk SNP carriership of rs12122721 [A] in brain tissue was assessed in order to analyze whether this corresponds with mRNA expression levels. SNP detection was performed using Taqman assay (Applied Biosystems) as previously described [13]. NDC, MS and AD donors were all in Hardy Weinberg equilibrium (p = 0.72) and the call rate was 98.9%.

### Immunohistochemistry

Immunohistochemistry was performed on frozen sections of brain tissue to detect kif21b protein. Briefly, 6 μm frozen sections were cut and thawed onto gelatine-chrome alum coated glass slides. Slides were kept overnight at room temperature in humidified atmosphere and air-dried for 1 h. Thereafter, slides were fixed in acetone containing 0.02% (v/v) H_2_O_2_. Slides were then air-dried for 10 min, washed with PBS and incubated with optimally diluted primary antibody specific for kif21b. Incubation with secondary antibody was performed, followed by tyramide signal amplification (TSA) according to the manufacturers’ protocol (Invitrogen). HRP activity was revealed by incubation for 10 min at RT with 3-amino-9-ethyl-carbazole (AEC, Sigma) substrate, leading to a bright red precipitate. Slides were embedded with glycerol-gelatin (Boom). Incubation with isotype control antibodies of irrelevant specificity and omission of primary antibody were used as negative controls.

### Grey matter lesion staging

MS grey matter brain lesions were staged on the basis of demyelination criteria, as described earlier [14]. Grey matter tissue was stained with monoclonal antibodies against PLP, NeuN and MAP2 using immunohistochemical detection. The slices were scanned using a Nanozoomer microscope (Hamamatsu). Lesions were digitally staged according to the classification scheme for grey matter demyelinating lesions, using the Nanozoomer Digital Pathology software version 1.2. Shortly, lesions were scored as mixed white-grey matter (Type I), intracortical (Type II), subpial (Type III) and lesions stretching from the pia mater until the border of white matter (Type IV). Two independent observers (KLK, MvM), blinded to clinical information and kif21b expression, scored grey matter lesions based on PLP staining, on a standardized scoring form. Morphometric information regarding total area of the slide, area containing white matter in the tissue (based on absence of MAP2 and NeuN expression), total number, types and area of grey matter lesions was assessed. Moreover, expression of HLA-II was scored as well as meningeal infiltration. In addition, when the slide contained white matter lesions, the area and HLA-II expression of these lesions was scored.

### Staging of MS white matter lesions

MS brain lesions were staged based on internationally accepted criteria for inflammation and demyelination as described earlier [15],[16]. We used three different markers, i.e. acid phosphatase, HLA-DP,DQ,DR and neutral lipids. To detect infiltrating myeloid cells, we stained acid phosphatase (lysosomal enzyme). Activated cells can be detected with an antibody against HLA-DP,DQ,DR. Myelin breakdown products, reflecting active demyelination, were detected with oil-red O (marker for neutral lipids).

### Immunofluorescence

Double-labelling of kif21b expressing cells with NeuN, MAP-2, HLA-II or CD68 was performed using immunofluorescence as described previously [17]. Sections were first incubated with primary antibody mixture for 1 h, followed by incubation of a mixture of secondary antibodies for 30 min. Sections were mounted in Prolong Gold (Molecular Probes) with 80 ng/ml DAPI (Molecular Probes).

Confocal fluorescence images were obtained on a Leica SP5 confocal system (Leica Microsystems CMS GmbH), equipped with an Argon laser (488 nm), Diode lasers (405 nm) and HeNe laser (633 nm). Images were taken using a 63x NA 1.4 objective. Images were recorded using standard Las-AF software version 2.6.3 (Leica Microsystems CMS GmbH). Possible crosstalk between the different fluorochromes, which could give rise to false-positive co-localization of the signals, was completely avoided by careful selection of the imaging conditions. Emission windows for Alexa Fluor 488 dyes were between 498-578 nm and for Alexa Fluor 647 between 643-775 nm, and Kalman averaging was used.

All antibodies used in this study are indicated in Additional file 1: Table S1b.

### Astrocytoma cell line cultures

U251MG human astrocytoma cells (ECACC 89081403, passage 28) were cultured in Dulbecco’s modified Eagle's medium (DMEM) with GlutaMAX/Ham's F-10 Nutrient Mix 1:1 containing 10% fetal bovine serum (FBS), 10 U/ml penicillin G and 10 mg/ml streptomycin (1% P/S) (all Invitrogen) at 37°C/5% CO_2_.

### Primary human astrocyte isolation and culture

For primary human adult astrocyte cultures, we obtained freshly dissected post-mortem subcortical white matter from a 79-year-old female control with a post-mortem delay of <18 hours (h) and a pH of the cerebrospinal fluid of 6.30 from the NBB. The tissue was collected in 25 ml cold Hibernate A (Invitrogen), and mechanically dissociated into small pieces. The tissue was digested with 0.2% trypsin (Invitrogen) and 0.1% DNAse I (Invitrogen) at 37°C, while shaking for 30 min. Next, 2 ml FBS was added to the mixture and, subsequently, the cells were collected by centrifugation. The pellet was taken up in DMEM without phenol red containing 10% FBS, 2.5% Hepes, and 1% P/S (all Invitrogen), and the suspension was filtered through a 60 μm mesh screen. Then, Percoll (Amersham/GE Healthcare) was added (half of cell suspension volume), and this mixture was centrifuged to separate cells, debris and myelin at 3220 relative centrifugal force (rcf) at 4°C for 30 min. The second layer (glial cell containing fraction) was collected and washed with complete DMEM (containing 10% FBS, 1% P/S, 2.5% Hepes and 1% gentamycin, all Invitrogen). After centrifugation, the pellet was taken up in complete DMEM and cells were seeded in a 6-cm uncoated culture dish. Microglia will adhere to the dish and the astrocytes will be present in the medium. After 6 h at 37°C/5% CO_2_, the medium, containing astrocytes, was taken off, centrifuged, and the microglia depleted pellet was seeded onto poly-L-lysine coated wells (PLL, Sigma-Aldrich, 15 μg/ml in PBS, 1 h at room temperature) in DMEM/Ham's F12 GlutaMAX medium containing 5% FBS and 1% P/S (all Invitrogen).

### Cytokine treatment in astrocytic cultures

U251MG astrocytoma cells or primary human astrocytes were plated in 24-well plates (25.000 cells per well) in their respective culture medium and allowed to adhere overnight. Then, medium was replaced and cells were cultured in medium with vehicle (PBS) or medium with a combination of recombinant human IFN-γ (R&D systems, 50 ng/ml) and recombinant human IL-1β (R&D systems, 50 ng/ml). After 48 h, cells were collected for mRNA isolation.

### Statistical analysis

Gene expression data and presence of the SNP compared to kif21b expression levels were analyzed using Kruskal Wallis test for multiple groups. For subgroup comparisons, Dunn’s Multiple Comparisons test was used. Mann Whitney U-test was performed to test differences between two groups. To determine correlations, non-parametric Spearman’s test was used. For survival analysis, the time between onset of MS and reaching EDSS 6.0 [18] was calculated and kif21b expression was dichotomized with expression above and below the median. Kaplan-Meier curves were constructed and differences in time to reach EDSS 6.0 were tested with a log-rank test. Hazard regression was performed to correct survival data for confounding variables. Statistical analysis was performed using SPSS version 20 (IBM). Graphs were made in GraphPad Prism and Dunn’s multiple comparisons test were also performed in GraphPad Prim version 5.04 (GraphPad Software Inc.). p-values <0.05 were considered statistically significant and are denoted in the figures as *p < 0.05, **p < 0.01 and ***p < 0.001.

## Results

### Characteristics of AD and MS patients and NDC

We assessed the size of the investigated tissue and the percentage of grey matter in the tissues of MS, AD and NDC and these characteristics were similar (Additional file 1: Figure S1). No significant differences in kif21b expression were found between tissues obtained from the medial temporal gyrus or the superior frontal gyrus (Additional file 1: Figure S2). The age at death, pH of the CSF, and post-mortem delay (PMD) were significantly different between NDC, MS and AD (Table 1). Linear regression showed no clear trend for kinesin expression and PMD or pH, in contrast to age at death (Additional file 1: Table S2). Thus, for disease specific kinesin expression, stratification according to age at death was performed based on the age quartiles of NDC.

**Table 1:** **Clinical and demographical data**

	NDC (n = 58)	MS (n = 50)	AD (n = 50)	p-value
Age at death (SD)	71 (13)	63 (13)	66 (9)	0.004
Median post-mortem delay in hours (IQR)	7:35 (6:18-9:16)	6:57 (5:25-8:15)	5:20 (4:27-6:13)	4*10^-7^
pH (IQR)	6.64 (6.45-6.94)	6.50 (6.36-6.71)	6.47 (6.34-6.67)	0.02
Percentage female	72	74	76	0.92
Kif21b rs12122721 genotype n (%)				
GG	27 (46.6)	31 (62.0)	21 (42.0%)	0.24
AG	26 (44.8%)	15 (30.0)	26 (52.0%)	
AA	3 (5.2%)	4 (8.0%)	3 (6.0%)	
Undetermined	2 (3.4%)	0 (0)	0 (0)	

IQR: interquartile range.

### Kif21b is increased in AD patients compared with MS and NDC

**Figure 1:**
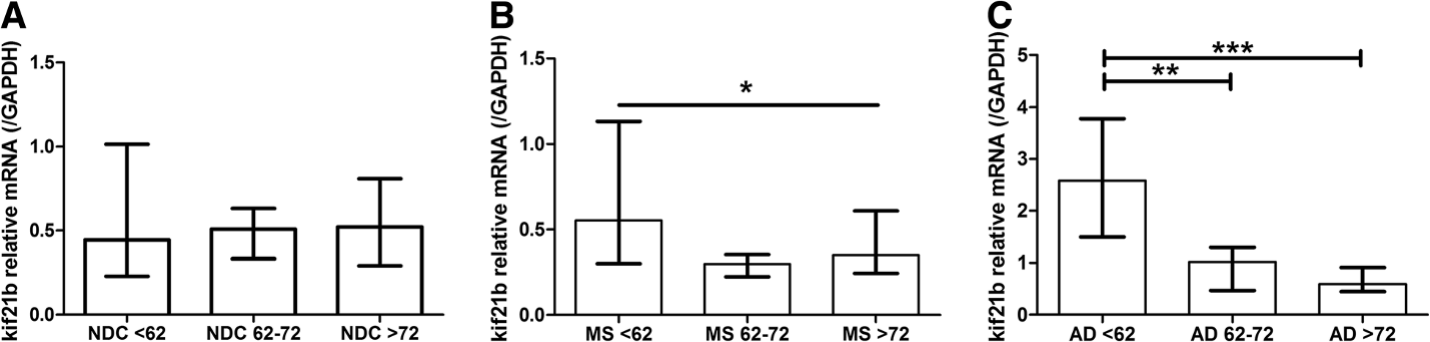
**Kif21b expression in the grey matter does not change during physiological ageing, but is significantly increased in young Alzheimer patients.** Kif21b expression was stratified according to the age at death into three categories based on age at death of the NDC (<25^th^ percentile represents <62 yrs, between 25-75^th^ percentile equals 62-72 yrs or >75^th^ percentile is >72 yrs). Kif21b was compared between the three age categories in **A)** NDC, **B)** MS and **C)** AD.

**Figure 2:**
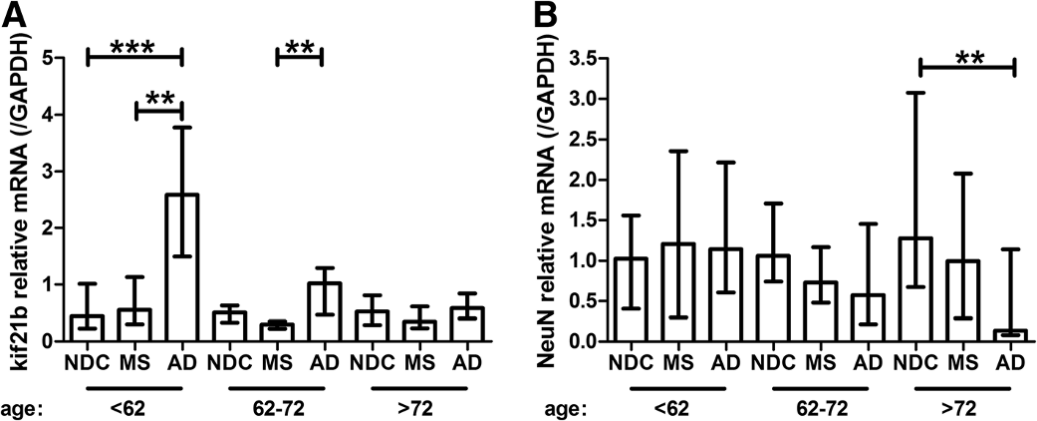
**Cortical kif21b is significantly increased in younger Alzheimer patients compared with MS and NDC. A)** Kif21b expression is significantly increased in AD patients younger than 62 years at death compared with MS and NDC younger than 62 yrs and in AD patients between 62-72 yrs compared with MS patients in the same age category. **B)** No significant differences were found in the expression of NeuN, a neuron specific marker, in the youngest two age categories. However, NeuN expression significantly decreased in elderly AD patients compared with NDC.

During physiological aging in NDC, no changes in kif21b expression were observed with increasing age (p = 0.9, Figure 1A). However, a significant decrease of kif21b over age was observed in MS (p = 0.03, Figure 1B) and AD patients (p = 5*10^-4^, Figure 1C). Comparing MS, AD and NDC in the different age groups, we found that kif21b is significantly increased in AD compared with MS patients and NDC. Interestingly, in the age category below 62 years of age, an approximately five-fold increase in kif21b was observed in AD patients compared with MS. Kif21b in AD compared to NDC was approximately six-fold increased (p = 9*10^-5^), whereas no significant differences were found between MS and NDC. In the age group from 62-72 years, a three-fold increase in AD compared with MS and two-fold between AD and NDC (p = 0.005) was observed. In the elderly patients (>72 years), no differences between MS, AD and NDC were found (p = 0.23, Figure 2A). Next, we assessed whether the alterations in kif21b expression might reflect differences in neuron density. Therefore, we used NeuN (RBFOX3) as marker for neuron density and found a significant reduction in NeuN in the AD patients compared with NDC above 72 years of age (p = 0.006, Figure 2B), whereas no differences between these groups in kif21b expression were found (Figure 2A). Additionally, we determined the expression of other CNS specific markers, GFAP (astrocytes) and MBP (oligodendrocytes). In the youngest AD, we observed a significant increase of GFAP (p = 0.02) compared with MS and in MBP (p = 0.003) compared with NDC and MS (Additional file 1: Figure S3). No significant differences between males and females were observed or between primary progressive and secondary progressive MS (Additional file 1: Figures S4 and S5 respectively). Additionally, we validated kif21b expression levels corrected for PMD as there were slight, though significant differences in PMD between the different patients groups. Similar results were found for kif21b expression corrected for PMD as with the uncorrected kif21b expression, indicating that it is unlikely that the PMD has influenced the kif21b expression (Additional file 1: Figure S6).

### Kif21b in spinal cord correlates with cortical kif21b expression of MS patients

The medial temporal gyrus is preferentially affected in AD, while this area is affected in approximately 34% of clinically isolated syndrome patients (CIS, the first attack of neurological deterioration in MS) [1]. To investigate whether kif21b levels in a commonly affected area in MS correlates with expression in the temporal gyrus, we assessed kif21b expression in seven matched brain and spinal cord tissues. We found a clear trend between expression in brain and spinal cord in MS patients (p = 0.06, Additional file 1: Figure S7).

### No correlation between increased kif21b expression and kif21b risk SNP

**Figure 3:**
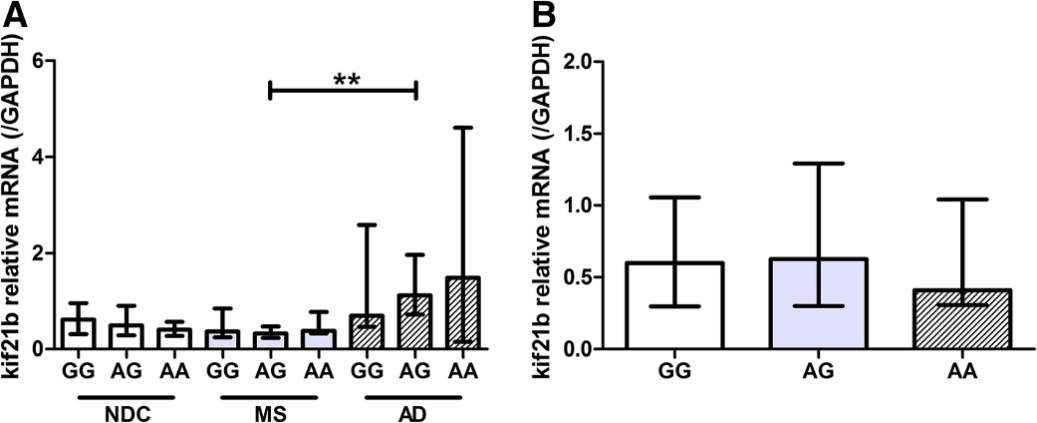
**No differences between MS risk genotypes and kif21b expression levels. A)** Kif21b expression was compared within NDC, MS and AD and stratified according to kif21b rs12122721 [A] risk genotype and within genotypes between the different diseases. No obvious differences between the risk genotypes or diseases were observed. **B)** The three groups of donors were pooled and kif21b expression was stratified according to the rs12122721 genotypes. No significant differences were observed between the three genotypes.

We hypothesised that kif21b expression is influenced by the kif21b risk SNP and therefore kif21b expression was stratified according to rs12122721 [A] risk allele carriership in the three patients groups. In none of the three groups, carriership of the risk allele correlated with kif21b expression (all p > 0.54, Figure 3A). Even after pooling all donors for the three groups, no difference in kif21b expression between the different genotypes was found (p = 0.74, Figure 3B).

### More severe pathology is associated with increased kif21b expression in AD and MS

**Figure 4:**
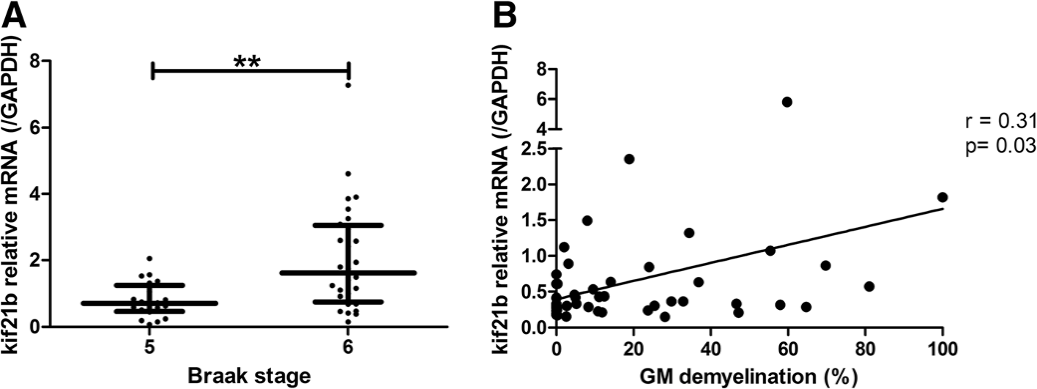
**Kif21b expression correlates with both AD and MS pathology. A)** Kif21b expression was stratified for AD patients according to the Braak criteria. AD patients with more severe AD pathology (Braak stage 6) had significantly higher kif21b expression levels. **B)** For MS patients, the total area of grey matter demyelination was quantified and percentage of cortical demyelination was calculated. With increasing percentage of GM demyelination, enhanced kif21b expression was observed.

Next, we hypothesised that kif21b expression correlates with the severity of neuropathology in Alzheimer and MS. AD pathology was staged according to the Braak criteria [12] by experienced neuropathologists from the Netherlands Brain Bank. Kif21b mRNA expression was stratified according to these criteria. Kif21b expression is significantly higher in Braak stage 6 compared with Braak stage 5 (p = 0.003, Figure 4A). Additionally, we assessed whether the level of kif21b expression correlated with the extent of grey matter demyelination in MS patients. A significant correlation between kif21b and the percentage of cortical demyelination was found (Spearman’s rho = 0.31, p = 0.03, Figure 4B), indicating that kif21b is increased with more severe neuropathology.

### Kif21b is approximately ten-fold more abundant in MS white matter compared to NDC

**Figure 5:**
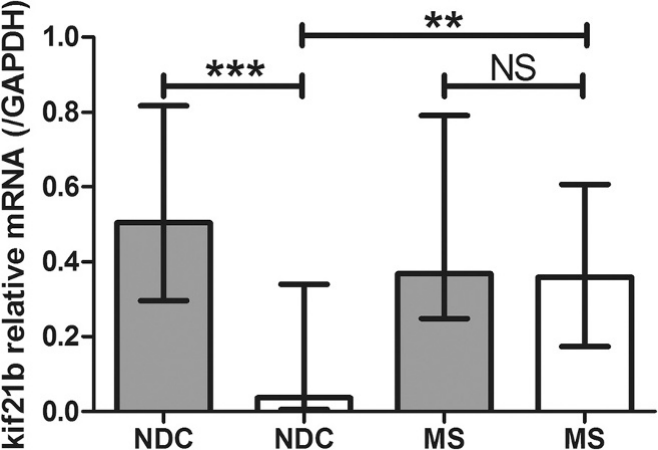
**In MS white matter, kif21b is approximately ten-fold increased compared with NDC white matter.** Kif21b expression in the white matter of 16 MS and 11 NDC was determined. MS patients had approximately ten-fold increased kif21b expression compared with NDC (white bars). As a reference, the kif21b cortical expression levels are shown (grey bars). In MS patients, kif21b white matter expression equalled kif21b grey matter expression, whereas in NDC kif21b expression in the white matter compared with the grey matter was significantly lower.

In view of the significant correlation between the amount of grey matter demyelination and kif21b expression, we hypothesised that kif21b is increased in the white matter (WM) of MS patients, a common area of demyelination in MS. Kif21b mRNA expression was assessed in the white matter of 11 NDC and 16 MS patients (Additional file 1: Table S3) and we found that kif21b was approximately ten-fold increased in MS compared with NDC (median and IQR of kif21b expression relative to GAPDH in NDC 0.037, 0.006-0.34 vs. MS 0.36, 0.17-0.34, p = 0.03, Figure 5). Comparing the levels of expression between the white and grey matter in NDC revealed that kif21b is highly expressed in the grey matter and hardly in the WM as expected with a kinesin highly enriched in dendrites of neurons. Interestingly, in MS patients the levels of expression in the WM and GM are comparable (Figure 5). Both GFAP and MBP were increased in MS patients (Additional file 1: Figure S8).

### Kif21b protein is increased in AD patients and expressed in neurons and astrocytes

**Figure 6:**
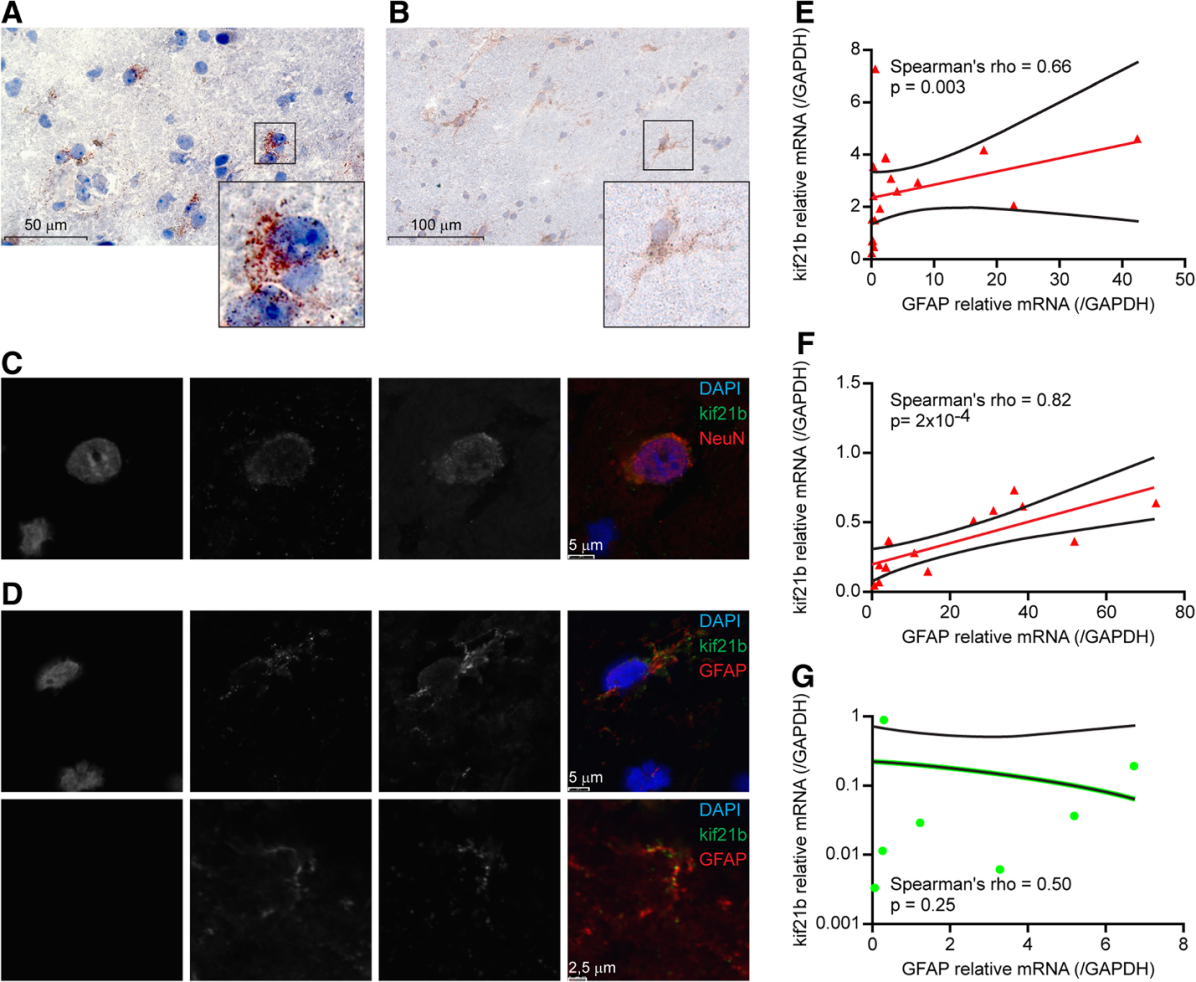
**Kif21b is expressed in astrocytes and costains with GFAP in AD grey matter and MS white matter.** Representative immunohistochemical staining of kif21b expression in **A)** the grey matter (out of 25 investigated tissues, Table 2) and **B)** in the white matter (out of 26 samples, Additional file 1: Table S4). Based on morphology, different cell types express kif21b. Immunofluorescence staining in six tissues revealed that **C)** kif21b is expressed in the somata of neurons and **D)** kif21b is expressed in the cell body as well as the processes of astrocytes. For both C and D, the left column is DAPI, the middle column kif21b and the right column the cell-type specific marker as indicated in the merged figure. Kif21b expression correlates with GFAP expression **E)** in the young AD patients in the grey matter, **F)** in MS white matter, **G)** no correlation was found between GFAP and kif21b in white matter of NDC.

Using immunohistochemistry, we next assessed whether kif21b protein is also increased in the cortex of AD patients compared with NDC and MS (Additional file 1: Table S3) to validate our mRNA findings in grey matter tissues. Firstly, we assessed which cell types expressed kif21b. Both glia cells and neurons expressed kif21b (Figure 6A). Next, we semi-quantitatively scored kif21b expression in age-, gender- and genotype matched NDC, MS and AD patients. Kif21b expression was higher in AD patients compared with MS and NDC, both in neurons and in glia cells (Table 2), confirming the mRNA data (Figure 2). Next, we assessed whether the kif21b protein was also expressed in the white matter. In one NDC out of 16, we could not detect any kif21b protein in the white matter, in three out of 14 MS patients kif21b protein was not observed in the WM (Figure 6B). In the WM of three AD patients, we also observed kif21b protein expression. The WM expression of kif21b is highly variable within individual tissues, some areas had very high kif21b expression, whereas other regions are completely kif21b negative (Additional file 1: Table S4) and this hampers reliable quantification. This variation possibly indicates that kif21b is expressed in activated cells. Lastly, we assessed which cell types expressed kif21b. No kif21b expression was found in microglia cells (Additional file 1: Figure S9A and B) or in SMI32 positive axons (Additional file 1: Figure S9C and D). High kif21b expression was found as expected in neurons (Figure 6C) and interestingly also in astrocytes (Figure 6D). Only in young AD patients, GFAP mRNA correlated with kif21b expression in the grey matter (Figure 6E, Additional file 1: Figure S10). In frontotemporal dementia, especially young patients have reactive astrocytosis [19], comparable with our findings. Additionally, also in the white matter of MS patients (Figure 6F), but not NDC (Figure 6G), a positive correlation between GFAP and kif21b mRNA expression was found, again pointing to activation of astrocytes.

**Table 2:** **Quantification of kif21b expression in situ**

Patient id	Age at death	Gender	Presenting symptom	Time to EDSS 6.0 (years)	Neuropathology assessment		Kif21b^1^		Kif21b genotype
					Braak stage	Amyloid	Neu-rons	Glia cells	
NDC2	49	F	NA	NA	0	ND	+	-	GA
NDC4	53	F			0	0	+/-	-	GA
NDC6	56	F			0	0	+/-	-	GG
NDC8	62	F			1	0	+/-	-	GA
NDC10	70	F			0	B	+	+	GA
NDC13	50	M			1	0	-	-	AA
NDC29	77	M			1	B	+/- / +	-	GG
NDC30	78	M			1	A	+/-	+/-	AA
NDC36	82	M			1	A	+/-	-	GG
MS4	50	F	Myelitis	7	ND	ND	-	-	GG
MS6	56	F	ND	4^*^	ND	ND	+	+	GG
MS8	63	F	Optic neuritis	17	0	0	+	-	GA
MS10	70	F	Cerebrum	30	1	0	-	-	GG
MS23	56	M	Myelitis	2	ND	ND	-	-	AA
MS25	57	M	Myelitis	6	ND	ND	+/-	-	GG
MS40	74	M	Myelitis	8	3	0	-	-	AA
AD2	57	F	NA	NA	6	C	+	+/-	GA
AD4	57	F			6	C	+	-	GG
AD6	59	F			5	C	+/++	+/++	GA
AD8	63	F			5	C	+/-	-	GG
AD10	70	F			5	C	+	-	GA
AD13	42	M			6	C	+/-	+/-	AA
AD15	54	M			6	C	+	+/-	AA
AD20	58	M			4	C	++	++	GG
AD42	76	M			5	C	+	+/-	GA

In age-, gender- and genotyped matched MS and AD patients, kif21b protein expression was assessed. NA not applicable, ND not determined or documented.

^1^scored as: - no positive cells, +/-1-2 positive cells per field, + maximum of ~30% of the cells positive, ++ ~60% of the cells positive, +++ ~80% positive cells and +++ (virtually) all cells positive.

^*^time to EDSS 9.0.

Neuropathology assessment based on Braak criteria [12].

### Kif21b is upregulated during astrocyte activation

**Figure 7:**
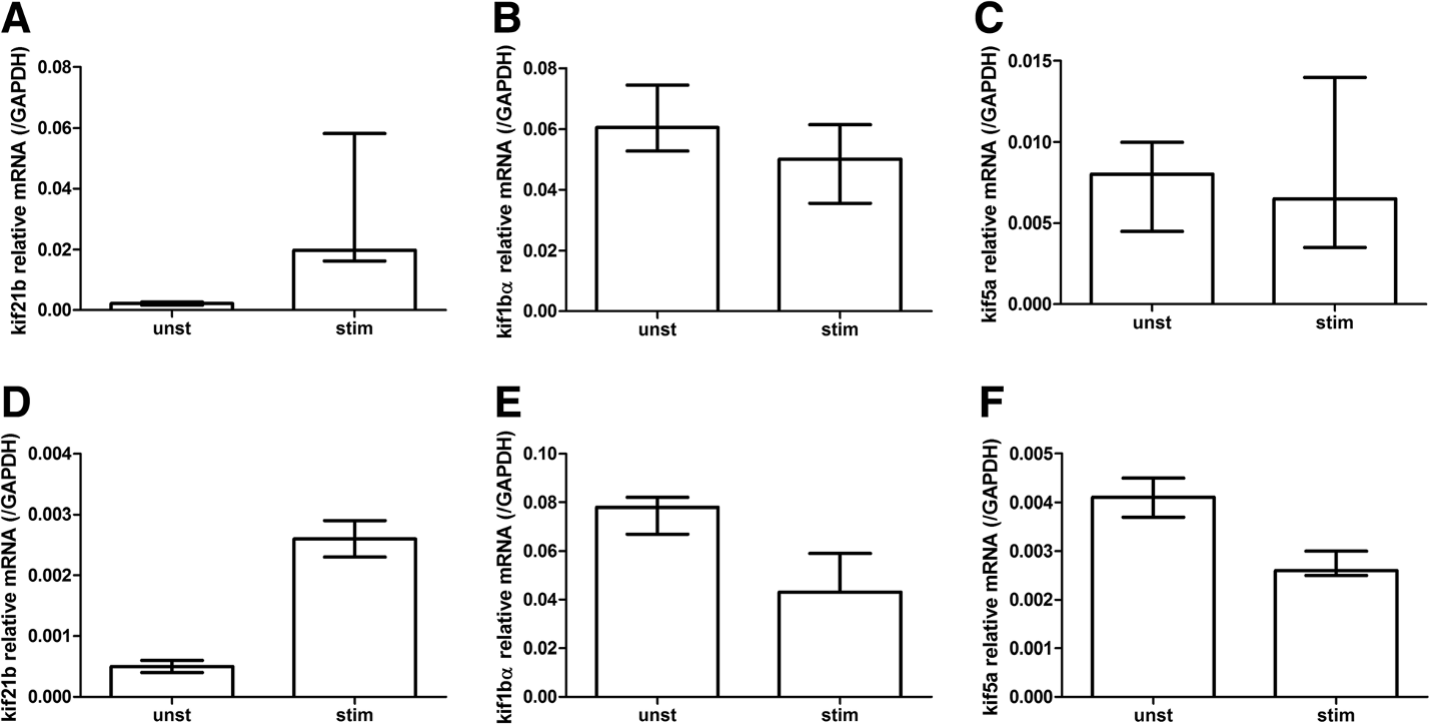
**Expression of kif21b, but not other kinesins increases upon astrocyte activation.** The astrocytoma cell line U251 was stimulated with IL-1β and IFN-γ for 48 h in three independent experiments in duplo. **A)** Kif21b expression increased approximately nine-fold upon stimulation, whereas **B)** kif1bα did not change **C)** and kif5a slightly decreased. Next, primary isolated astrocytes of a NDC were also stimulated in one experiment in triplo and similar changes in kinesin expression were found **(D-F)**.

Most knowledge on kifs focused on neurons and virtually nothing is known regarding kinesins in astrocytes. Therefore, we decided to determine kif21b in astrocytes further. We asked whether astrocytes express a basal level of kif21b or that the observed kif21b expression in both WM and GM astrocytes reflects reactive astrocytosis. The astrocytoma cell lines U251 was stimulated with IL-1β and IFN-γ for 48 h and kif21b expression was compared between unstimulated and stimulated conditions. The activation status was determined by assessing IL-6 production (Additional file 1: Figure S11A and B). Upon astrocyte activation, kif21b increased approximately nine-fold (Figure 7A). To assess whether this is specific for kif21b or that other kinesins are also induced and therefore reflecting cell activation in general, kif1bα and kif5a expression levels were also determined. The expression of kif1bα did not change during astrocyte activation (Figure 7B), whereas kif5a slightly decreased (Figure 7C). Lastly, we determined whether this mechanism is also observed in primary astrocytes isolated directly post-mortem, to corroborate the U251 study. Similar, but more pronounced changes in kinesin expression upon activation were observed (Figure 7D-F).

### High levels of kif21b expression are associated with a more rapid and severe disease course in MS and AD

**Figure 8:**
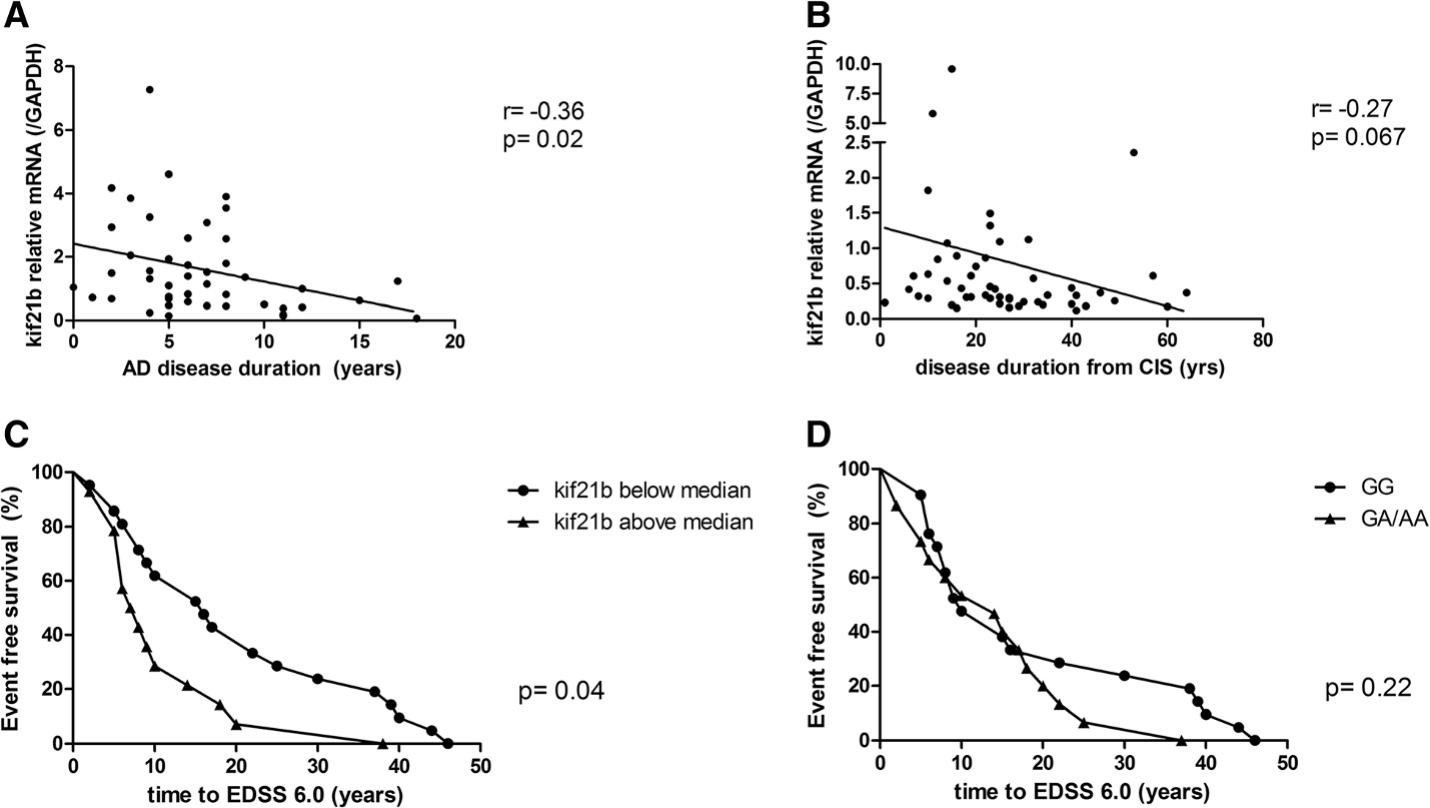
**Abundant kif21b expression is associated with a shorter disease duration and accelerated progression to sustained neurological disability.** Kif21b expression was correlated with the disease duration of **A)** Alzheimer patients and **B)** multiple sclerosis patients. In both diseases, shorter disease duration was associated with abundant kif21b expression. **C)** Kif21b expression in MS patients was dichotomised for the expression above the median and below the median and survival analysis was performed in MS patients. MS patients with kif21b expression above the median had a significantly shorter time to develop EDSS 6.0, the neurological score for sustained disability. **D)** The time to develop EDSS 6.0 was stratified according to kif21b MS risk SNP carriership. The rs12122721 [A] SNP is not associated with accelerated time to develop EDSS 6.0.

Given the correlation between kif21b and neuropathology, we hypothesised that kif21b expression contributes to more rapid progression in neurodegeneration. Therefore, we assessed whether a correlation between disease duration and kif21b expression existed. In AD, high kif21b expression was associated with a significantly shorter disease duration (Spearman’s rho -0.36, p = 0.02, Figure 8A) and a similar trend was observed in MS (Spearman’s rho -0.27, p = 0.067, Figure 8B). Next, we assessed whether kif21b levels in MS also correlated with a shorter time to reach EDSS 6.0, a measure for sustained disability. Kif21b was dichotomised as expression above or below the median and a Kaplan Meier survival curve was constructed. Kif21b expression above the median is associated with an approximately two-fold more rapid progression to EDSS 6.0 compared to expression below the median (Log Rank test, p = 0.04, Figure 8C). MS patients with abundant kif21b expression had a three-fold increased risk to have an accelerated disease course (hazard ratio (HR) for abundant kif21b in the development of sustained disability corrected for age at onset 3.0, 95% CI 1.4-6.4, p = 0.003), independent of gender. Given the significant correlation between kif21b and the percentage of GM demyelination, we assessed whether the time to develop EDSS 6.0 is independent of GM demyelination*.* Abundant kif21b expression adjusted for the percentage of GM demyelination revealed that a rapid progression to EDSS 6.0 is independent of the percentage of GM demyelination. Moreover, GM demyelination is not associated with the time to develop EDSS 6.0 (Additional file 1: Table S5). No significant difference in time to reach EDSS 6.0 between kif21b risk SNP carriers and non-risk SNP carriers (p = 0.22, Figure 8D) was observed, indicating that the accelerated neurodegeneration is independent of the MS risk SNP (HR 1.3, 95% CI 0.61-2.79, p = 0.07). Additionally, adding the kif21b risk SNP to the Hazard regression model for the effect of abundant kif21b expression on the time to develop EDSS 6.0 did not alter the results, indicating that kif21b expression is the explanatory factor (Additional file 1: Table S5). Given the association between astrocyte activation and kif21b expression, we assessed whether high expression of GFAP correlated with an accelerated time to EDSS 6.0 and found no association (HR 0.97, 0.45-2.07, p = 0.94). Thus, abundant kif21b expression is an independent predictor for accelerated progression to develop EDSS 6.0 (Additional file 1: Table S5).

### Other kinesins are not associated with accelerated neurodegeneration

Lastly, we assessed whether abundant kinesin expression in general is associated with a more rapid neurodegeneration in MS. Therefore, we tested six other kinesins of different kinesin families. Two of these kinesins have previously been associated with MS (kif1b and kif5a), although subsequent validation of kif1b failed [20], whereas the other kinesins are unrelated to MS. Members of the kinesin-1 superfamily (kif5a, b, c and the kinesin light chain, KLC) are associated with AD*.* For kif5a specifically, we observed a trend that abundant expression is associated with a shorter time to develop EDSS 6.0 (HR 2.0, 95% CI 0.97-4.2, p = 0.06). None of the other kinesins showed a trend towards accelerated neurodegeneration (Additional file 1: Table S6), indicating that specific kinesins are involved in a more rapid disease course.

## Discussion

Genetic variations in the kif21b locus confer a modestly increased risk to develop MS [3]. However, the underlying mechanisms are currently completely unknown. We here show that abundant levels of kif21b are associated with accelerated progression to EDSS 6.0 and a shorter disease duration in MS. Similarly in AD, high levels of kif21b expression are found in patients who have had a short disease duration. Moreover, increased levels of kif21b are associated with more severe neuropathology in both AD and MS. The observed differences in expression and progression were independent of the MS risk SNP. Similar to our findings, Harding et al. found no association between the kif21b SNP and the time to develop EDSS 6.0 [21]. We and others have functionally investigated the intronic rs12122721 [A] SNP, whereas recently the rs7522462 [G] SNP was reported in the largest MS GWAS. Rs7522462 is located in an open reading frame nearby the kif21b gene. These two polymorphisms are in linkage disequilibrium with the each other (R^2^ > 0.73, based on http://www.broadinstitute.org/mpg/snap/). Therefore, it is unlikely that the assessment of the previously MS-associated SNP in this study instead of the most recently reported SNP in the kif21b locus has affected the results.

Genetic variations in both kif21a and kif21b are linked to several human diseases. Microduplications in the kif21b locus were identified in two patients with neurodevelopmental disorders. These two patients had delayed motor and cognitive development [22]. Kif21a is a family member of kif21b and has similarities in amino acid composition, but its expression pattern is different. Kif21a is expressed throughout neurons and kif21b is mainly expressed in dendrites. Kif21a is linked to congenital fibrosis of the extraocular muscles type 1 (CFEOM1), a disease characterised by absence of motor neurons of the midbrain and in the superior division of the oculomotor nerve [23]. Moreover, CFEOM1 with a Marcus Gunn jaw-winking phenomenon is associated with another kif21a mutation [24]. Variation in the kif21b locus is also strongly associated with ankylosing spondylitis (M. Bechterew) [25] and moderately with ulcerative colitis [26]. This suggests that kif21b has an additional function in the immune system, which is beyond the scope of the current report.

Also other kinesin family members are genetically associated with human diseases. For example, a variant in kif5a locus has been linked with the risk to develop rheumatoid arthritis [27] and is a candidate SNP in MS [28]. Moreover, kif5a mutations are associated with the development of hereditary spastic paraplegia (SPG10), a neurodegenerative disease. At least one mutation in kif5a decreases both the anterograde and retrograde transport flux of neurofilaments [29]. Interestingly, we observed a trend that abundant kif5a is also associated with a more rapid development of sustained disability in MS (Additional file 1: Table S6). Additionally, a SNP in kif1b has been implied as risk SNP for MS [30], but a subsequent study failed to replicate this finding [20].

Kif21b, a plus end-directed motor kinesin, is produced in the cell body of the neuron, after which it is transported to the dendrites, where it is mainly expressed. Kif21b might be involved in delivering currently unknown cargoes to the distal regions of dendrites [5]. The physiological function of kif21b and the mechanisms by which abundant kif21b and possibly kif5a in neurons contribute to accelerated neurodegeneration remain to be determined. Whether abundant kif21b is the cause or consequence of the accelerated neurodegeneration needs to be investigated.

Increasingly, it has been recognised that in addition to white matter lesions, also grey matter pathology in MS occurs already in early in the disease [2]. One of the major pathological changes besides demyelination in MS is axonal damage, however relatively little is known about neuronal damage [31]. It is intriguing to speculate that kinesins might play a pivotal role in these processes as they are important for axonal transport and thereby for neuron integrity, function and survival. Currently, as far as we are aware of, hardly anything is known about the expression and distribution of kinesins in MS or AD. Additionally, we showed that kif21b is also expressed in activated astrocytes Abundant levels of kif21b expression have been linked to a poorer prognosis of several forms of cancers. Increased kif21b expression was observed in gliomas, brain tumours with a very poor prognosis, especially the glioblastoma multiforme with a survival rate of less than a year [32]. Increased kif21b levels were also observed in three well-characterised astrocytoma cell lines, KINGS-1, no. 11 and Becher compared with benign gliomas or healthy brain tissue [33]. This is comparable to our findings that kif21b expression increases during astrocyte activation. High levels of kif21b expression in neurodegenerative diseases are associated with a more rapid disease course as we have shown here, again pointing to an important role of kif21b in astrocytes. The mechanistic consequences of abundant kif21b expression and the upregulation of kif21b upon activation in astrocytes remain speculative as the function of kinesins in general and kif21b specifically in astrocytes is currently unknown.

It is interesting that more severe AD pathology is associated with enhanced kif21b expression. Reactive astrocytes are found in regions with amyloid beta deposits in AD pathology and these astrocytes have accumulated neuronal amyloid beta 42 [34]. Astrocytes can be activated when co-cultured with microglia cells in the presence of amyloid beta [35]. Additionally, astrocytosis is mainly observed in younger frontotemporal dementia patients, and this is supportive for our findings regarding the correlation between kif21b and GFAP only in the young AD patients [19]. Astrogliosis is observed in MS white matter lesions, whereas GFAP expression in grey matter lesions is not increased [36]. This supports the correlation between GFAP and kif21b in WM of MS patients, but not in cortical demyelination (Figure 6F and Additional file 1: Figure S10). The role of astrocytes in the neurodegenerative component of multiple sclerosis is also increasingly being recognised [37].

Further research on the cargo transported by kif21b and the exact function of this kinesin will be important to gain more insight into the exact mechanisms whereby kif21b contributes to neurodegeneration. Understanding the function of kif21b in neurons and astrocytes, may shed new light on possible mechanisms to therapeutically target neurodegeneration and malignant brain tumours.

## Conclusions

Kif21b is significantly increased in AD patients compared with MS and NDC. In both MS and AD patients, upregulated kif21b expression was associated with more severe neuropathology. In MS patients, abundant kif21b expression was associated with accelerated neurodegeneration and a shorter time to develop EDSS 6.0. In AD patients, increased kif21b was associated with a shorter disease duration. Kif21b expression was found in neurons and astrocytes, upon activation of astrocytes, kif21b significantly increased.

## Additional file

## Electronic supplementary material

Additional file 1:**Six supplementary tables and 11 supplementary figures.**(DOCX 2 MB)

Below are the links to the authors’ original submitted files for images.

Authors’ original file for figure 1

Authors’ original file for figure 2

Authors’ original file for figure 3

Authors’ original file for figure 4

Authors’ original file for figure 5

Authors’ original file for figure 6

Authors’ original file for figure 7

Authors’ original file for figure 8

## Abbreviations

AD: Alzheimer's disease
ALS: Amyotrophic lateral sclerosis
APP: Amyloid precursor protein
CFEOM1: Congenital fibrosis of the extraocular muscles type 1
CI: Confidence interval
CIS: Clinically isolated syndrome
dpi: Days post infection
EAE: Experimental autoimmune encephalomyelitis
GFAP: Glial fibrillary acidic protein
GWAS: Genome wide association study
HLA-II: Human leukocyte antigen class II
HR: Hazard ratio
IFN-γ: Interferon gamma
IL-1β: Interleukin 1 beta
IQR: Interquartile range
kif: Kinesin family member
MAP2: Microtubule-associated protein 2
MBP: Myelin basic protein
MS: Multiple sclerosis
NDC: Non-demented control
NeuN: Neuronal nuclear antigen
NFT: Neurofibrillary tangles
PLP: Myelin proteolipid protein
PMD: Post-mortem delay
SNP: Single nucleotide polymorphism
