# Supplementary material for: Abundant kif21b is associated with accelerated progression in neurodegenerative diseases
Source: Acta Neuropathol Commun. 2014 Oct 3;2:144. doi: 10.1186/s40478-014-0144-4 (PMC4207309; doi:10.1186/s40478-014-0144-4)
Supplement: Supplementary file 1 — Additional file 1:Six supplementary tables and 11 supplementary figures.(DOCX 2 MB) [file 40478_2014_9144_MOESM1_ESM.docx]

**Abundant kif21b is associated with accelerated progression in neurodegenerative diseases.**

**Supplementary information**

Karim L. Kreft MD, PhD ^1,2,3^, Marjan van Meurs ^2,3^, Annet F. Wierenga-Wolf ^2,3^, Marie-Jose Melief ^2,3^, Miriam E. van Strien PhD ^4,6^ , Elly M. Hol PhD ^4,5,6^, Ben A. Oostra PhD ^7^, Jon D. Laman PhD ^2,3^ and Rogier Q. Hintzen MD, PhD ^1,2^

Depts. ^1^ Neurology, ^2^ Immunology, ^3^ MS Center ErasMS and ^7^ Clinical Genetics Erasmus MC, University Medical Center, Rotterdam, The Netherlands; ^4^ Astrocyte Biology & Neurodegeneration, Netherlands Institute for Neuroscience, An Institute of the Royal Academy of Arts and Sciences, Amsterdam, The Netherlands, ^5^ Swammerdam Institute for Life Sciences, Center for Neuroscience, University of Amsterdam, The Netherlands, ^6^ Dept. Translational Neuroscience, University Medical Center Utrecht, Utrecht, The Netherlands

Corresponding author:

Prof. Rogier Q. Hintzen, MD, PhD

Erasmus MC, Department of Neurology

Room Ba 4.92

P.O. box 2040

3000 CA Rotterdam

The Netherlands

Phone: +31-10-7033780, Fax: +31-10-7035927

[r.hintzen@erasmusmc.nl](mailto:r.hintzen@erasmusmc.nl)**Supplementary Table 1.** *Primers and antibodies used in this study*

n.s.

| **Supplementary table 1a***. Primers* | | | | | |
| --- | --- | --- | --- | --- | --- |
| Gene | Forward primer | | | Reverse primer | |
| Kif1a | cttggcgacatcactgacat | | | gctggacagggctgagag | |
| Kif1bα | GCATTAAAGGCTGTTTTACTACCG | | | GTAAGGCACTGGGGCACA | |
| Kif1bβ | GAAGCTGGAGCTCCTACATGA | | | ACGAGTCGCTCAGGGATTT | |
| Kif1c | gttcattcgggagcaacact | | | gctccagagtgaccaccact | |
| Kif5a | acacgagaagagcaccaagc | | | gactgctcatgtcgctcgta | |
| Kif5b | CTGAAGACGCAAATGTTGGA | | | TGCATATTGTCTTGATCCCTTC | |
| Kif21b | GGCTGGACCTGAGTTCAAAG | | | GGTCAAGGATCTCCTCGTTG | |
| NeuN | GGGGAACCCCTACACCAA | | | GAATTCAGGCCCGTAGACTG | |
| GFAP | AGAGGGACAATCTGGCACA | | | CAGCCTCAGGTTGGTTTCAT | |
| MBP | AGCCCTCTGCCCTCTCAT | | | GGAGCCGTAGTGAGCAGTTC | |
| IL-6 | gatgagtacaaaagtcctgatcca | | | ctgcagccactggttctgt | |
| GAPDH | Commercial kit Applied Biosystems | | | | |
| GUSB | Commercial kit Applied Biosystems | | | | |
| **Supplementary table 1b***. Antibodies* | | | | | |
| **Marker** | **Clone** | **Isotype** | **Dilution** | | **Supplier** |
| Kif21b |  | rIgG | 1/25 | | Atlas |
| MAP2 | HSM5 | mIgG1 | 1/400 | | Pierce |
| PLP | plpc1 | mIgG1 | 1/2000-1/4000 | | Bioconnect |
| MOG | Z12 | mIgG2a | 1/200 | | Generous gift from S. Amor |
| HLA-II | CR3/43 | mIgG1 | 1/1600-1/20000 | | Dako |
| GFAP | ASTRO6 | mIG1 | 1/50*-1/200 | | Thermo Scientific |
| CD68 | EBM11 | mIgG1 | 1/200 | | Dako |
| NeuN | MAB377 | mIgG1 | 1/400*-1/6000 | | Chemicon |
| SMI-32 | SMI-32 | mIgG1 | 1/100*-1/400 | | Sternberger monoclonals |
| Secondary antibodies | | | | | |
| RAM-Ig-HRP | NA | rIg | 1/100 | | Dako |
| RAM-Ig-bio | NA | rIg | 1/400 | | Dako |
| SAV-AF488 | NA | NA | 1/100 | | Molecular Probes |
| GAM-Ig-AF594 | NA | GIg | 1/300 | | Molecular Probes |
| Isotype controls | | | | | |
| mIgG1 | MAB002 | - | Depends on primary antibody concentration | | R&D |
| mIgG2a | MAB003 | - | 1/358 | | R&D |
| Rabbit Ig | NA | - | 1/125 | | R&D |

* Antibody dilution used for immunofluorescence; NA, not applicable

**Supplementary Table 2.** *Age at death is a significant predictor for the levels of kif21b expression in AD patients, but not in MS or NDC.*

|  | pH CSF | | | PMD | | | Age at death | | |
| --- | --- | --- | --- | --- | --- | --- | --- | --- | --- |
| disease | Correlation coefficient (95% CI) | R^2^ | p-value | Correlation coefficient  (95% CI) | R^2^ | p-value | Correlation coefficient  (95% CI) | R^2^ | p-value |
| NDC | 0.012 | 2*10^-4^ | 0.93 | 0.090 | 0.008 | 0.52 | -0.067 | 0.005 | 0.63 |
| MS | -0.26 | 0.07 | 0.09 | 0.096 | 0.009 | 0.51 | -0.24 | 0.06 | 0.10 |
| AD | -0.27 | 0.07 | 0.06 | -0.088 | 0.008 | 0.55 | **-0.55** | **0.31** | **3*10^-5^** |

Correlation coefficients between kif21b expression and pH of the CSF, post-mortem delay and age at death were assessed by linear regression. Significant correlations are indicated in bold.

**Supplementary Table 3.** *Demographic characteristics of white matter donors included in this study.*

|  | NDC (n=18) | MS (n=23) | AD (n=3) |
| --- | --- | --- | --- |
| Age at death (SD) | 80 (6) | 59 (14) | 81 (4) |
| Female/ male (n) | 8/10 | 17/6 | 2/1 |
| Post-mortem delay in hours (IQR) | 7.0 (5.58-8.20) | 7.15 (5.40-8.32) | 4.15 (4.0-8.35) |
| pH CSF | 6.42 (6.20-6.83) | 6.54 (6.33-6.76) | 6.66 (6.04-8.28) |
| Age at onset (SD) | NA | 35 (8) | 74 (8) |

IQR, inter-quartile range

**Supplementary Table 4.** *Kif21b protein expression in the white matter is highly variable.*

|  | HLA-II | kif21b |  | MOG | kif21b |  | ORO | kif21b |
| --- | --- | --- | --- | --- | --- | --- | --- | --- |
| NDC1 | 0 | 0/3 |  | 4 | 0/3 |  | 0 | 0/3 |
| NDC2 | 1/2 | 2 |  | 4 | 0.5/2 |  | 0 | 0.5/2 |
|  | 0 | 0.5/1 |  |  |  |  |  |  |
| NDC3 | 0 | 0 |  | 4 | 0 |  | 0 | 0 |
| NDC4 | 2 | 2 |  | 4 | 0/2 |  | 0 | 0/2 |
|  | 0.5 | 0.5/1 |  |  |  |  |  |  |
|  | 0 | 0/1 |  |  |  |  |  |  |
| NDC5 | 2 | 2 |  | 4 | 0.5/2 |  | 0 | 0.5/2 |
|  | 3 | 0.5 |  |  |  |  |  |  |
| NDC6 | 2 | 0.5/2 |  | 4 | 0.5/2 |  | 0 | 0/2 |
|  | 0.5/1 | 0.5/1 |  | 2 | 1 |  |  |  |
| NDC7 | 0 | 0/2 |  | 4 | 0/2 |  | 0 | 0/2 |
| NDC8 | 1 | 0.5 |  | 4 | 0 |  | 0 | 0/2 |
|  | 2/3 | 0 |  | 2 | 0 |  |  |  |
|  | 2/3 | 2 |  |  |  |  |  |  |
| NDC9 | 0.5 | 0/2 |  | 2 | 1/2 |  | 0 | 0/2 |
|  |  |  |  | 4 | 0/2 |  |  |  |
| NDC10 | 0/0.5 | 0/2 |  | 4 | 0.5/1 |  | 0 | 0/2 |
|  |  |  |  | 2 | 1/2 |  |  |  |
|  |  |  |  | 1 | 2 |  |  |  |
| NDC11 | 0.5 | 1/2 |  | 0 | 0 |  | 0 | 0/2 |
|  | 0.5 | 0 |  |  |  |  |  |  |
| NDC12 | 0.5 | 0 |  | 4 | 0/2 |  | 0 | 0/2 |
|  | 0.5 | 1/2 |  |  |  |  |  |  |
|  |  |  |  |  |  |  |  |  |
| MS1 | 2/3 | 3 |  | 4 | 2/3 |  | 0 | 2/3 |
|  | 1 | 2/3 |  |  |  |  |  |  |
| MS2 | 2 | 0 |  | 0 | 0 |  | 3 | 2 |
|  | 3 | 2 |  | 4 | 1/2 |  | 3 | 0 |
|  | 3 | 0 |  |  |  |  |  |  |
| MS3 | 2 | 1/2 |  | 2 | 0.5/1 |  | 0.5 | 2 |
|  | 1 | 2 |  | 4 | 1/2 |  |  |  |
|  | 1 | 1/2 |  |  |  |  |  |  |
| MS4 | 3/4 | 0/2 |  | 4 | 0/2 |  | 0 | 0/2 |
|  | 0 | 0 |  | 1 | 0 |  | 2 | 0 |
| MS5 | 3 | 1/3 |  | 4 | 1/3 |  | 0 | 1/3 |
|  |  |  |  | 1 | 3 |  | 0 | 0 |
| MS6 | 1/2 | 0 |  | 1 | 0 |  |  |  |
|  |  |  |  | 4 | 0 |  |  |  |
|  |  |  |  | 3 | 0 |  |  |  |
| MS7 | 0/0.5 | 0 |  | 4 | 0 |  | 0 | 0 |
| MS8 | 1/2 | 0 |  | 4 | 0 |  | 0 | 0 |
| MS9 | 2 | 0/3 |  | 2/3 | 1/2 |  | 0 | 0/3 |
|  |  |  |  | 4 | 2/3 |  |  |  |
|  |  |  |  | 2/3 | 0 |  |  |  |
| MS10 | 0.5 | 0/2 |  | 3/4 | 0/2 |  | 0 | 0/2 |
|  | 2 | 2 |  |  |  |  |  |  |
| MS11 |  |  |  | 3 | 2/3 |  | 0 | 1/3 |
|  |  |  |  | 4 | 1/3 |  |  |  |
|  |  |  |  |  |  |  |  |  |
| AD1 | 0 | 0/3 |  | 4 | 0/3 |  | 0/0.5 | 0/3 |
| AD2 | 0 | 0.5/3 |  | 3 | 0.5/3 |  | 0 | 0.5/3 |
| AD3 | 1/2 | 0/3 |  | 1 | 0 |  | 0 | 0/3 |
|  |  |  |  | 4 | 0.5/2 |  |  |  |
|  |  |  |  | 4 | 3 |  |  |  |

Staining was scored as: 0= no positive cells, 0.5= 1-2 positive cells per field, 1= maximum of ~30% of the cells positive, 2= ~60% of the cells positive, 3= ~80% positive cells and 4= (virtually) all cells positive

**Supplementary Table 5.** *Abundant kif21b expression is an independent predictor for the time to develop EDSS 6.0 in MS*

| Variable | Hazard ratio (95% CI) | p-value | Additional variables in HR model | Adjusted HR (95% CI) | Adjusted p-value |
| --- | --- | --- | --- | --- | --- |
| Abundant kif21b expression | 2.2 (1.01-4.6) | 0.047 | Abundant kif21b expression | 3.0 (1.4-6.7) | 0.006 |
|  |  |  | Age at onset | 1.05 (1.02-1.08) | 0.004 |
|  |  |  | Abundant kif21b expression | 3.7 (1.6-8.7) | 0.003 |
|  |  |  | Age at onset | 1.06 (1.02-1.1) | 0.001 |
|  |  |  | GM demyelination | 1.01 (1.0-1.03) | 0.08 |
|  |  |  | Abundant kif21b expression | 2.99 (1.29-6.94) | 0.01 |
|  |  |  | Age at onset | 1.06 (1.02-1.10) | 0.005 |
|  |  |  | GFAP expression above median | 0.65 (0.27-1.55) | 0.33 |
|  |  |  | Abundant kif21b expression | 3.0 (1.3-6.7) | 0.009 |
|  |  |  | Age at onset | 1.05 (1.01-1.08) | 0.006 |
|  |  |  | MS risk SNP rs12122721 [A] | 1.13 (0.51-2.5) | 0.76 |
|  |  |  | Abundant kif21b expression | 3.4 (1.5-7.8) | 0.004 |
|  |  |  | Age at onset | 1.05 (1.02-1.08) | 0.004 |
|  |  |  | Gender | 1.8 (0.76-4.3) | 0.18 |
| GM demyelination | 1.01 (1.0-1.03) | 0.18 | GM demyelination | 1.01 (1.0-1.03) | 0.1 |
|  |  |  | Age at onset | 1.04 (1.01-1.08) | 0.02 |
| GFAP expression above median | 0.97  (0.45-2.07) | 0.94 | GFAP expression above median | 0.69 (0.30-1.58 | 0.38 |
|  |  |  | Age at onset | 1.04 (1.00-1.08) | 0.027 |

Abundant kif21b expression (defined as above or below the median expression) was adjusted for several variables in a Hazard regression model. Unadjusted and adjusted hazard ratios for time to develop EDSS 6.0 are indicated. Abbreviations: GM, grey matter; HR, hazard ratio; SNP, single nucleotide polymorphism

**Supplementary Table 6.** *Abundant expression of several other kinesins is not associated with accelerated development of sustained disability*

| Kinesin | HR* (95% CI) | p-value* | Previous neurological disease associations ^1^ | References |
| --- | --- | --- | --- | --- |
| Kif1a | 0.77 (0.37-1.6) | 0.48 |  |  |
| Kif1bα | 1.38 (0.63-3.02) | 0.43 | 1) MS GWAS, not validated  2) Charcot-Marie-Tooth disease  (human mutations, mouse model) | ([Aulchenko *et al.* , 2008](#_ENREF_1))  ([Booth *et al.* , 2010](#_ENREF_2))  ([Zhao *et al.* , 2001](#_ENREF_3)) |
| Kif1bβ | 0.97 (0.48-1.95) | 0.97 | 1) MS GWAS, not validated  2) Charcot-Marie-Tooth disease, both human mutations and mouse model |  |
| Kif1c | 0.94 (0.48-1.97) | 0.97 |  |  |
| Kif5a | 2.02 (0.97-4.2) | 0.06 | 1) MS candidate gene study  2) Kinesin-1 superfamily and kinesin-1 light chains implicated in transport of APP in AD  3) Hereditary spastic paraplegia (SPG10) | ([Alcina et al. , 2010](#_ENREF_4))  ([Muresan and Muresan, 2005](#_ENREF_5))  ([Reid et al. , 2002](#_ENREF_6))  ([Szpankowski et al. , 2012](#_ENREF_7)) |
| Kif5b | 1.41 (0.65-3.06) | 0.38 | Kinesin-1 superfamily and kinesin-1 light chains implicated in transport of APP in AD | ([Muresan and Muresan, 2005](#_ENREF_5))  ([Szpankowski *et al.* , 2012](#_ENREF_7)) |

Abundant expression of several other kinesins and the time to develop EDSS 6.0 was assessed in a Hazard regression model in MS patients.

* Adjusted for age at onset

^1^ Note, mutations in single patients or families are not taken into consideration in this table.

**Supplementary Figure 1.** *No significant differences in the size of the tissue or the percentage of white matter between MS, AD and NDC*


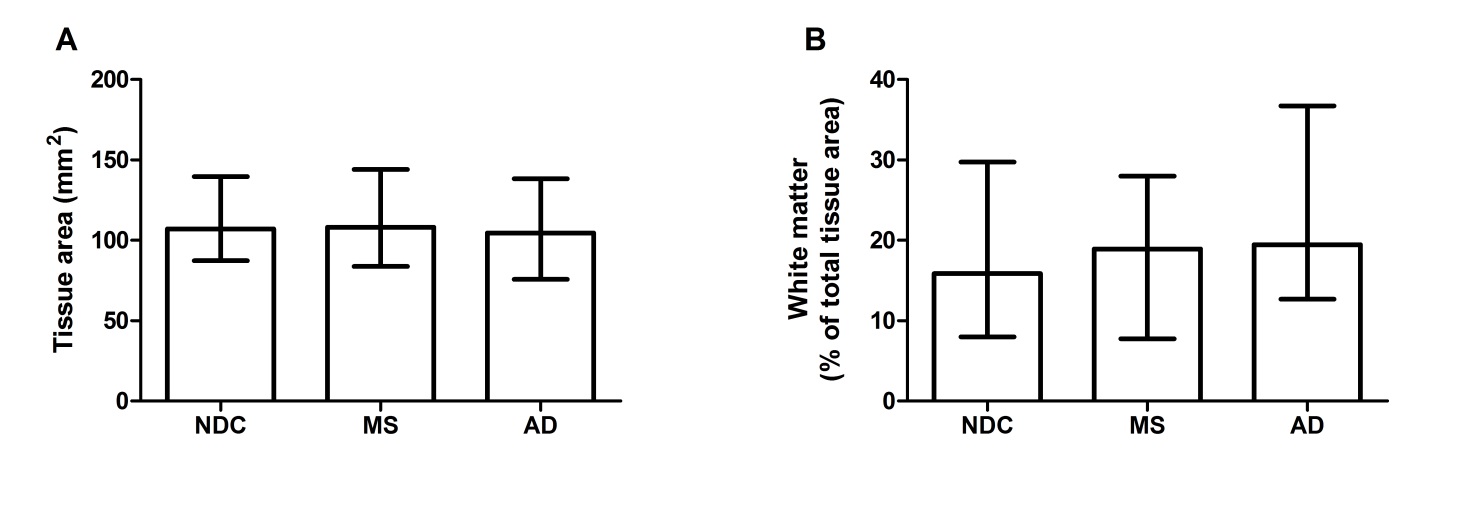


Morphometric parameters of the tissues investigated in this study were obtained using the NDC.view software (Hamamatsu). **A)** No significant difference was found in the total area of the tissue between 57 NDC, 49 MS and 50 AD (p=0.84). **B)** The percentage of white matter in the investigated tissues was calculated as percentage of the total tissue area. No significant differences were found between MS, AD and NDC (p=0.37). Statistics were calculated using a Kruskal-Wallis test.

**Supplementary Figure 2**. *No significant difference in kif21b expression in MS patients between the medial temporal gyrus and the superior frontal gyrus*


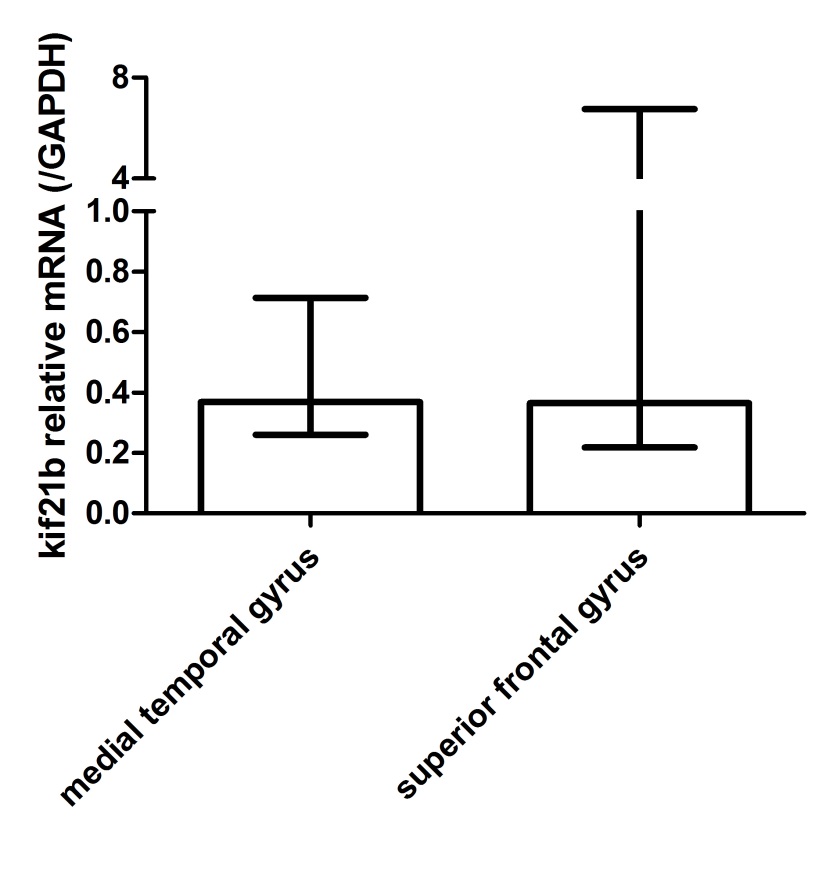


Kif21b expression was assessed in the medial temporal gyrus (n=44) and in the superior frontal gyrus (n=6). No significant regional differences were found (Mann Whitney U-test p=0.87).

**Supplementary Figure 3.** *GFAP and MBP mRNA are significantly increased in the younger AD patients*


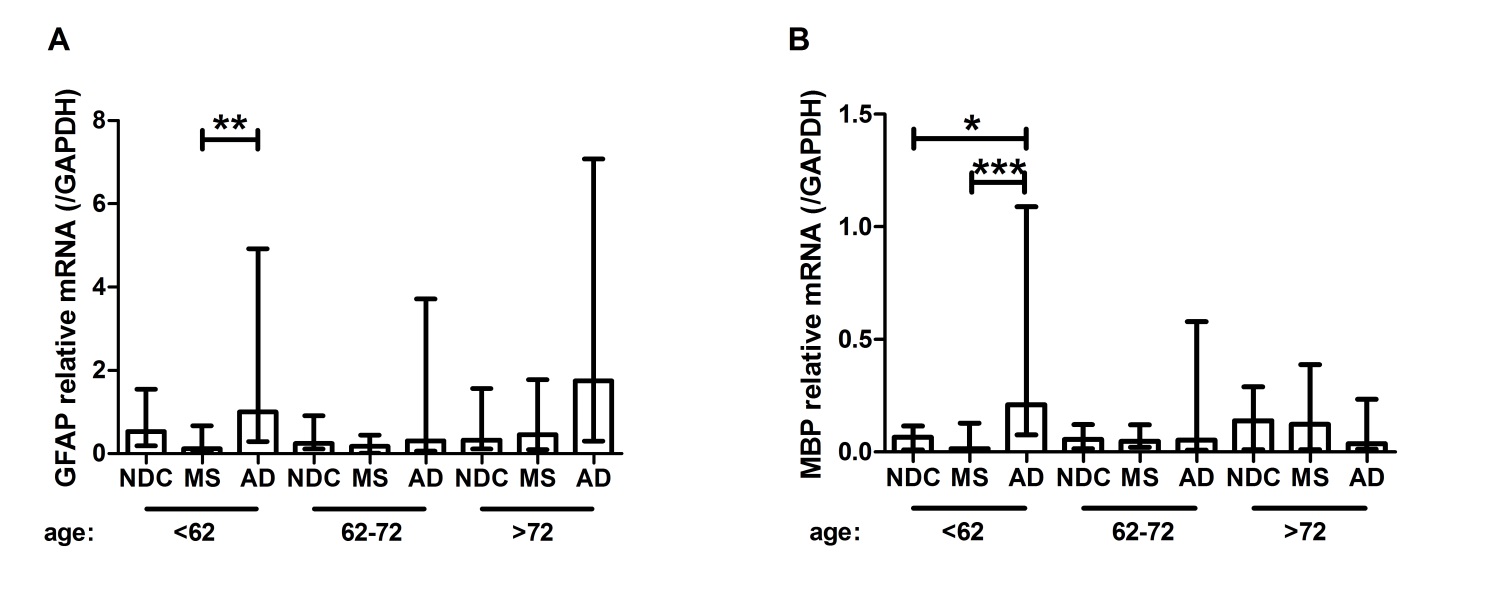


In the different age categories in NDC, AD and MS patients, the expression of **A)** GFAP and **B)** MBP was compared using a Kruskal-Wallis test with Dunn’s multiple comparison ad hoc analysis. For both GFAP and MBP, the variation in AD was large.

**Supplementary Figure 4.** *No differences in kif21b expression between males and females in the three donor groups*


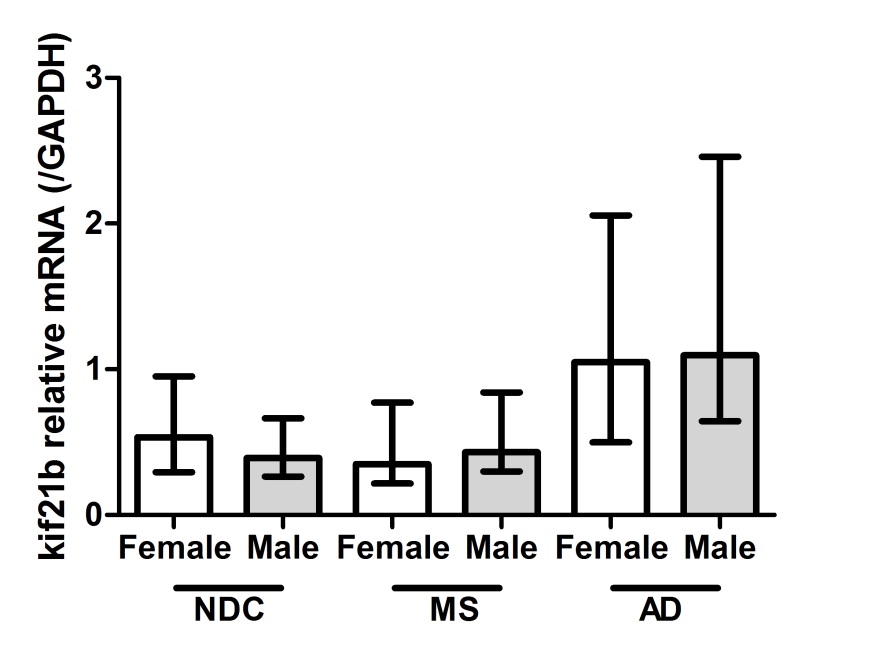


Since MS has a strong female predominance, we assessed whether kif21b expression was different between males and females. No significant gender differences were found in NDC, MS or AD (Mann Whitney U-test per disease, all p<0.26).**Supplementary Figure 5.** *No significant difference between distinct MS disease forms*


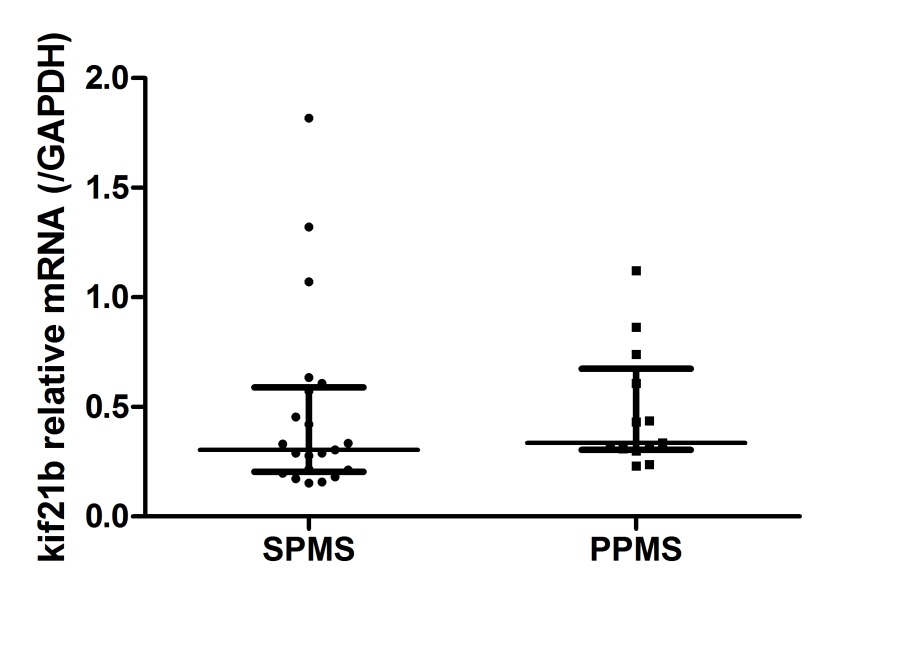


As we observed that abundant kif21b expression was associated with accelerated neurodegeneration, we assessed whether differences between primary progressive MS (PPMS, n=13) and secondary progressive MS (SPMS, n=21) exist. No significant difference in kif21b expression between SPMS and PPMS was found (Mann Whitney-U test, p=0.24).

**Supplementary Figure 6.** *Correcting kif21b expression for post-mortem gave similar results as uncorrected kif21b expression*


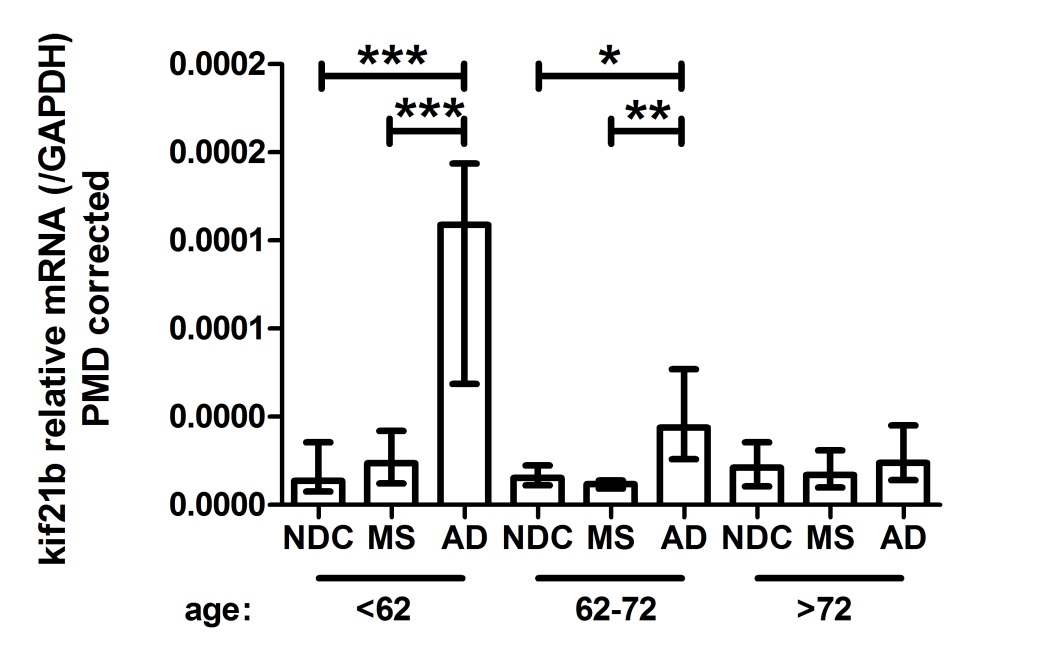


Since in AD patients, post-mortem delay (PMD) was a significant predictor for kif21b expression (Supplementary Table 2), we assessed whether correcting kif21b expression for PMD would alter the results. A similar pattern as in the main text Figure 2 was observed for the differences in kif21b expression between AD, MS and NDC in the different age categories. Statistics were calculated using a Kruskal-Wallis test with Dunn’s Multiple Comparison Test.

**Supplementary Figure 7.** *Kif21b expression levels are correlated in paired MS GM and spinal cord samples*


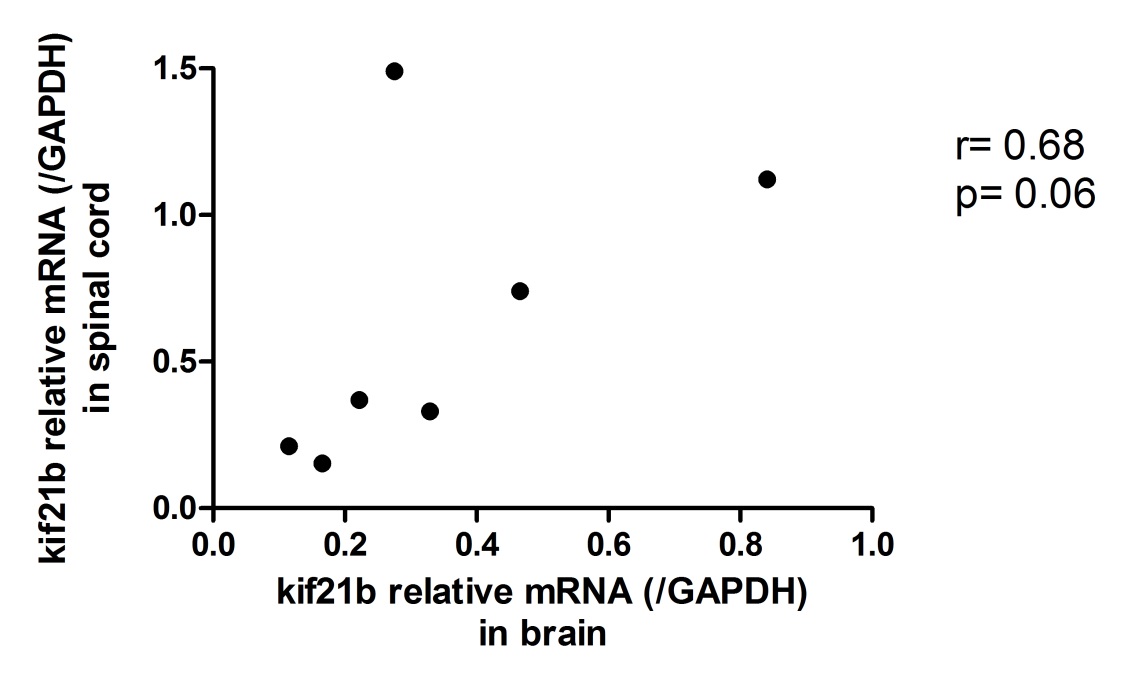


In paired grey matter and spinal cord samples (n=7), kif21b expression was determined. Kif21b expression in grey matter tissue and spinal cord samples gave similar results (p=0.06). Additionally, no differences between medial temporal and the superior frontal gyrus (Supplementary Figure 1) were found, indicating that the kif21b expression levels are probably not influenced by regional differences. Clincal and demographic details these paired GM and spinal cord samples are indicated in Supplementary Table 7.

**Supplementary Table 7.** *Clinical and demographic details of the paired grey matter and spinal cord samples.*

|  | Paired GM and spinal cord samples |
| --- | --- |
| Age at death (range) | 70 (44-82) |
| Female/ male (n) | 5/2 |
| Post-mortem delay in hours (range) | 7:45 (5:35-10:15) |
| pH CSF (range) | 6.40 (5.99-6.81) |
| Age at onset (range) | 36 (24-50) |

**Supplementary Figure 8.** *In MS white matter, MBP and GFAP expression are significantly increased*


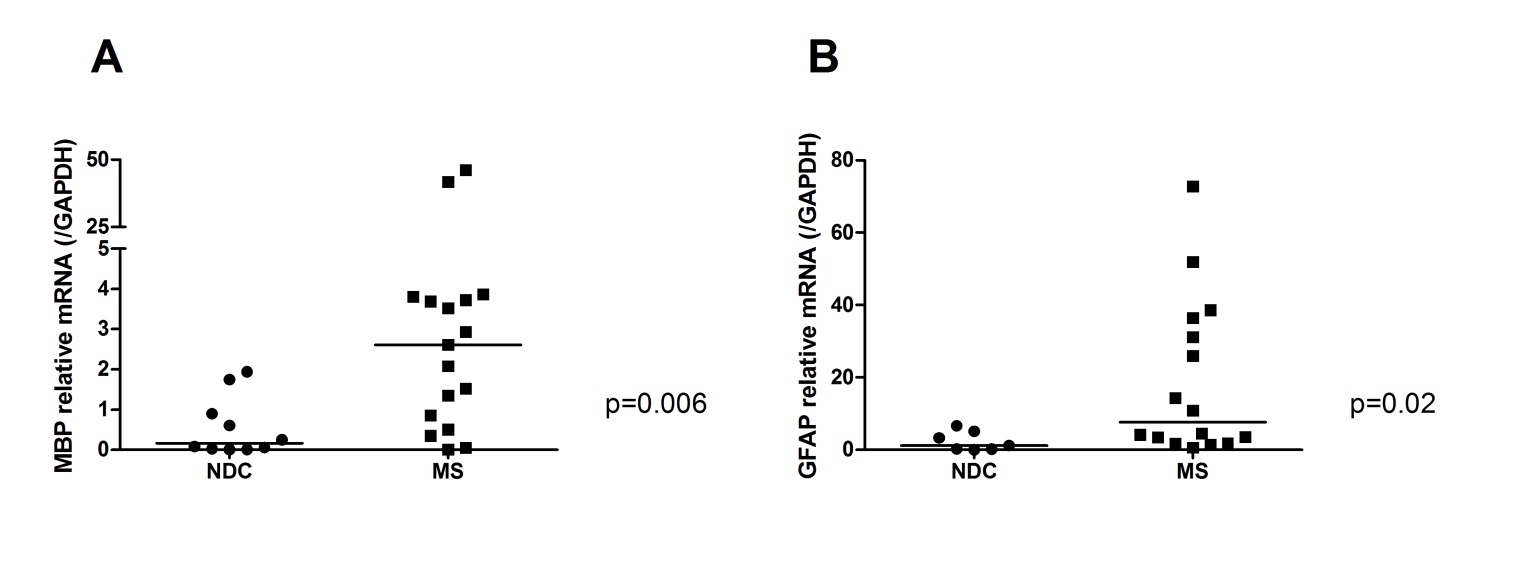


In the white matter of MS patients and NDC, the expression of **A)** MBP and **B)** GFAP was assessed. No significant difference between MS and NDC was found for NeuN (Fig. 6B, main paper). As expected, MBP was significantly increased, probably at least partially due to remyelination, and GFAP was slightly increased in MS patients, possibly due to astrogliosis. p-values were calculated using a Mann Whitney U-test.

**Supplementary Figure 9.** *Kif21b is not expressed in microglia cells or in SMI32 positive axons.*

*
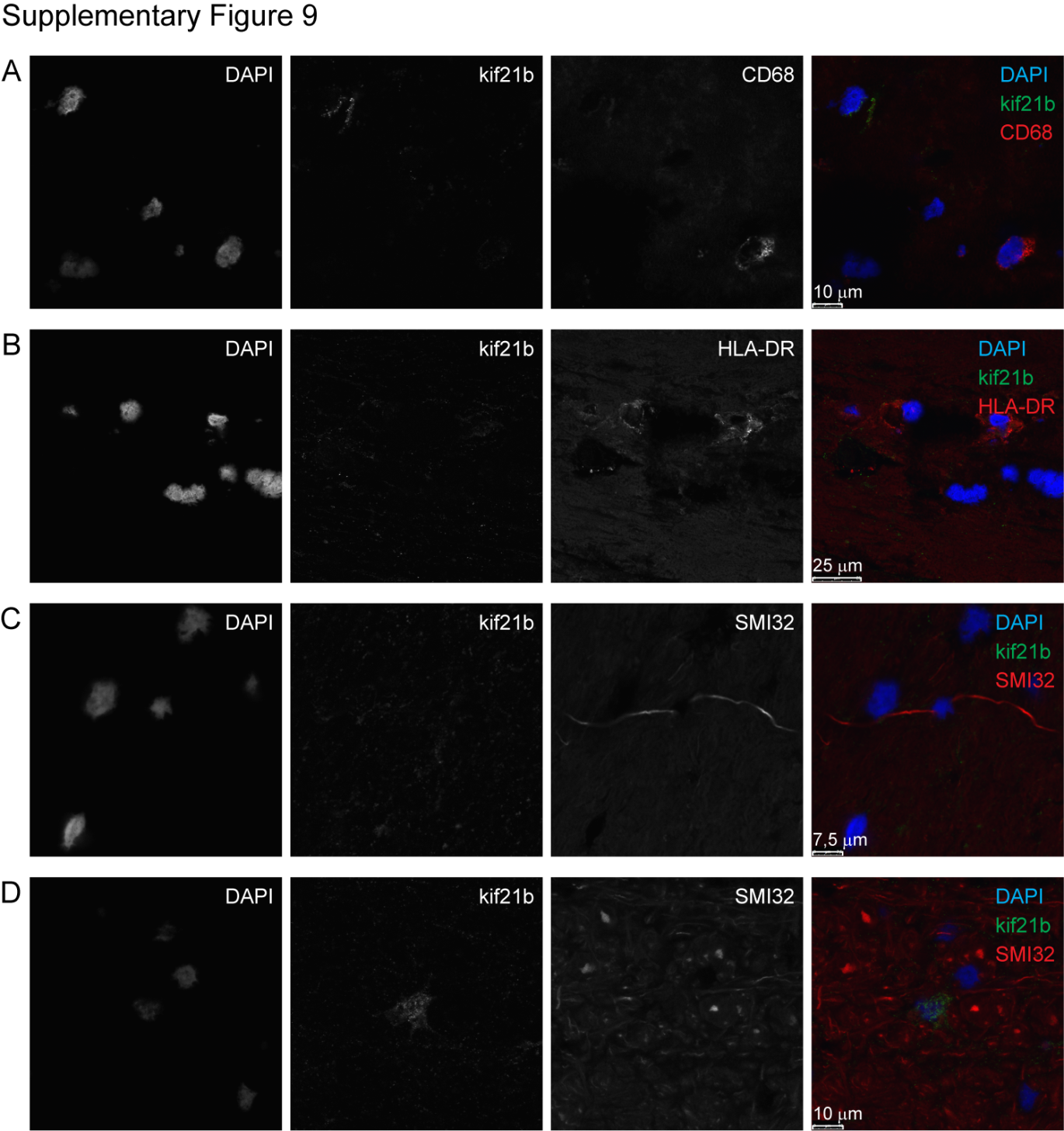
*

No kif21b expression was found in the white matter of in **A)** CD68 positive microglia/ macrophages or **B)** HLA class II positive cells. Additionally, no kif21b expression was observed in SMI32 positive axons **(C-D)**.

**Supplementary Figure 10.** *In NDC and MS patients and in elderly AD patients no correlation between cortical GFAP and kif21b expression exist.*


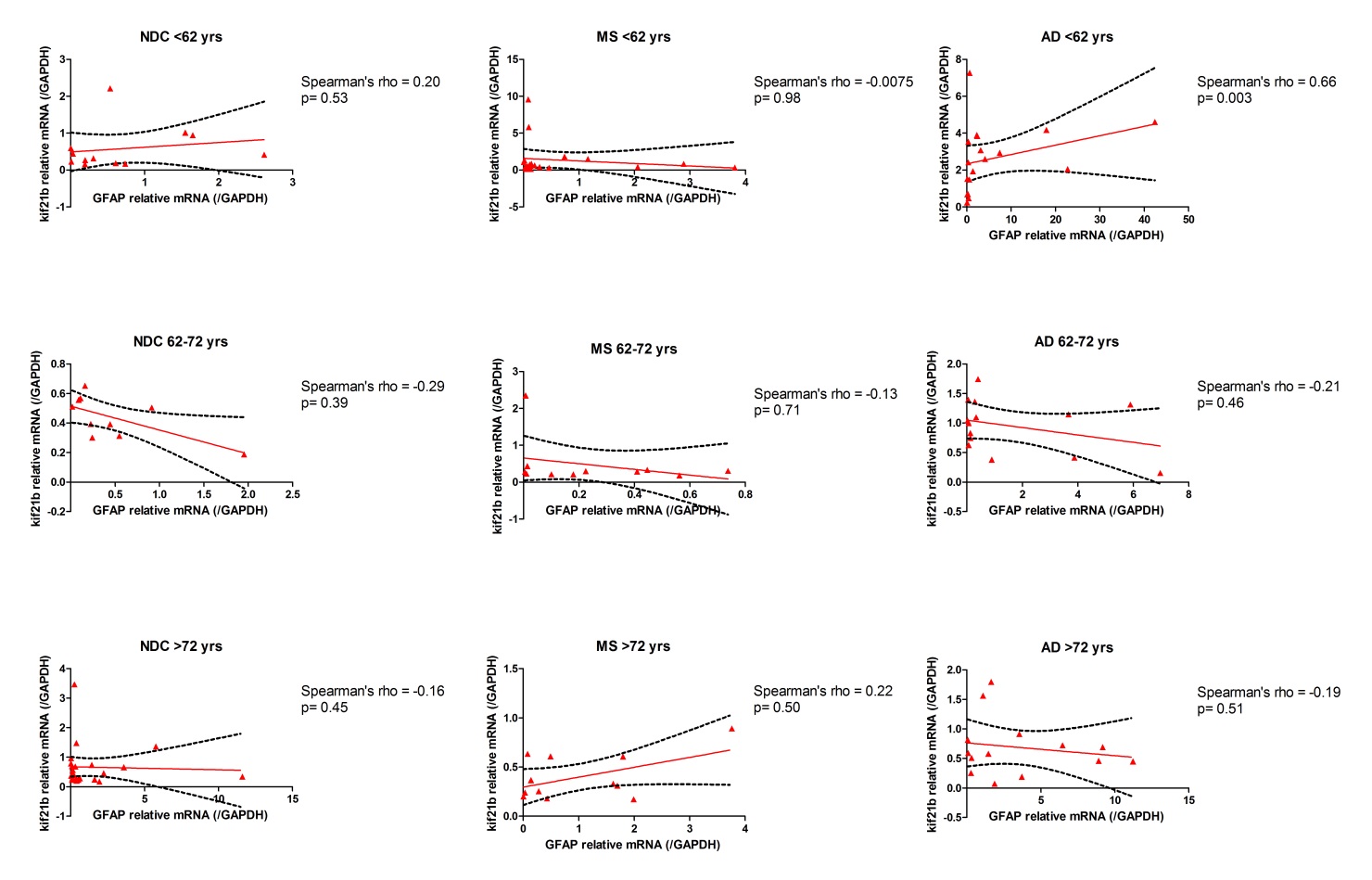


Correlation between kif21b and GFAP mRNA expression in the cortex of NDC (left column), MS (middle column) and AD (right column) stratified according to age categories < 62 years of age (upper row), 62-72 years (middle row) and above 72 years (lower row). Please note that the axes are different in the different figures. The graph for AD patients <62 years of age is also displayed in the main paper (Fig. 4E).

**Supplementary Figure 11.** *IL-6 increased upon astrocyte activation.*


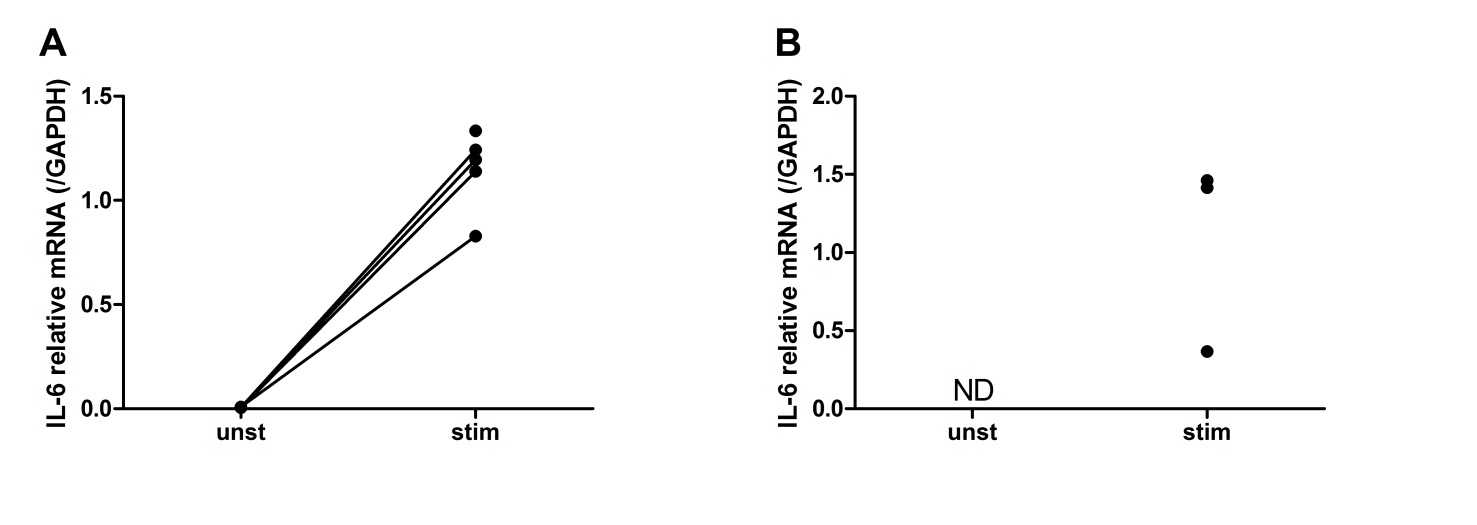


The U251 astrocytoma cell line **(A)** and primary astrocytes **(B)** were activated with IL-1β and IFN-γ for 48 h. IL-6 mRNA increased, indicating that the astrocytes are activated.

ND= not detected

**Supplementary references.**

Aulchenko YS, Hoppenbrouwers IA, Ramagopalan SV, Broer L, Jafari N, Hillert J, et al. Genetic variation in the KIF1B locus influences susceptibility to multiple sclerosis. Nature genetics. 2008 Dec;40(12):1402-3.

Booth DR, Heard RN, Stewart GJ, Cox M, Scott RJ, Lechner-Scott J, et al. Lack of support for association between the KIF1B rs10492972[C] variant and multiple sclerosis. Nature genetics. 2010 Jun;42(6):469-70; author reply 70-1.

Zhao C, Takita J, Tanaka Y, Setou M, Nakagawa T, Takeda S, et al. Charcot-Marie-Tooth disease type 2A caused by mutation in a microtubule motor KIF1Bbeta. Cell. 2001 Jun 1;105(5):587-97.

Alcina A, Vandenbroeck K, Otaegui D, Saiz A, Gonzalez JR, Fernandez O, et al. The autoimmune disease-associated KIF5A, CD226 and SH2B3 gene variants confer susceptibility for multiple sclerosis. Genes and immunity. 2010 Jul;11(5):439-45.

Muresan Z, Muresan V. Coordinated transport of phosphorylated amyloid-beta precursor protein and c-Jun NH2-terminal kinase-interacting protein-1. The Journal of cell biology. 2005 Nov 21;171(4):615-25.

Reid E, Kloos M, Ashley-Koch A, Hughes L, Bevan S, Svenson IK, et al. A kinesin heavy chain (KIF5A) mutation in hereditary spastic paraplegia (SPG10). American journal of human genetics. 2002 Nov;71(5):1189-94.

Szpankowski L, Encalada SE, Goldstein LS. Subpixel colocalization reveals amyloid precursor protein-dependent kinesin-1 and dynein association with axonal vesicles. Proceedings of the National Academy of Sciences of the United States of America. 2012 May 29;109(22):8582-7.
