# Supplementary figures and images for: Abundant kif21b is associated with accelerated progression in neurodegenerative diseases
Source: Acta Neuropathol Commun. 2014 Oct 3;2:144. doi: 10.1186/s40478-014-0144-4 (PMC4207309; doi:10.1186/s40478-014-0144-4)

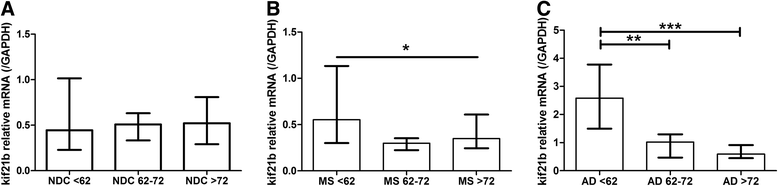

Supplement: Supplementary file 2 — Authors’ original file for figure 1 [file 40478_2014_9144_MOESM2_ESM.gif]

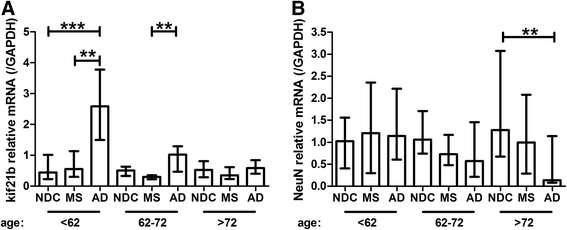

Supplement: Supplementary file 3 — Authors’ original file for figure 2 [file 40478_2014_9144_MOESM3_ESM.gif]

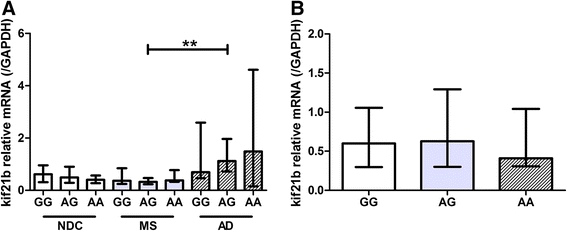

Supplement: Supplementary file 4 — Authors’ original file for figure 3 [file 40478_2014_9144_MOESM4_ESM.gif]

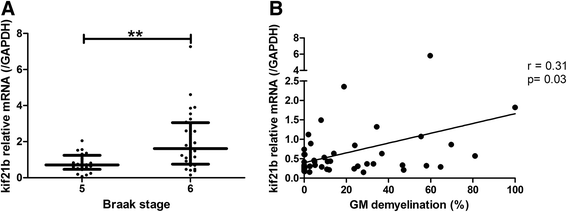

Supplement: Supplementary file 5 — Authors’ original file for figure 4 [file 40478_2014_9144_MOESM5_ESM.gif]

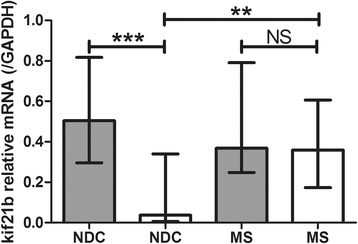

Supplement: Supplementary file 6 — Authors’ original file for figure 5 [file 40478_2014_9144_MOESM6_ESM.gif]

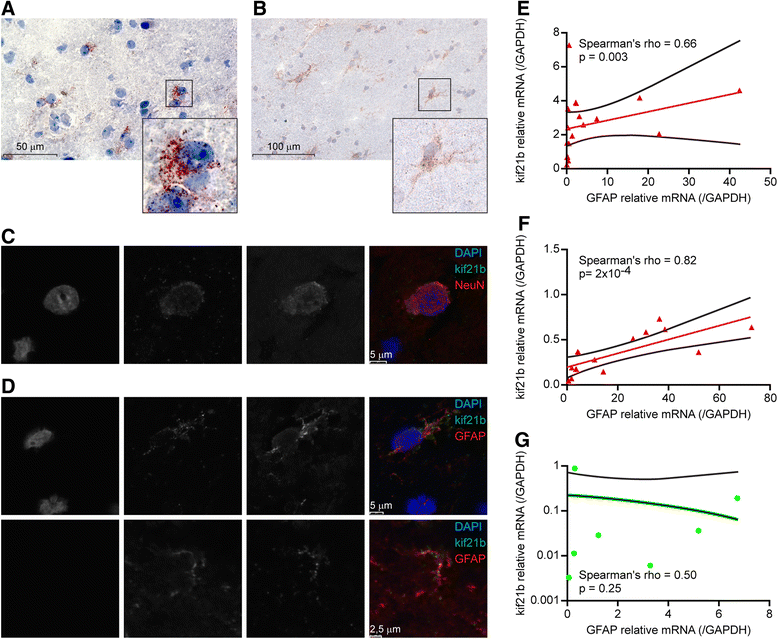

Supplement: Supplementary file 7 — Authors’ original file for figure 6 [file 40478_2014_9144_MOESM7_ESM.gif]

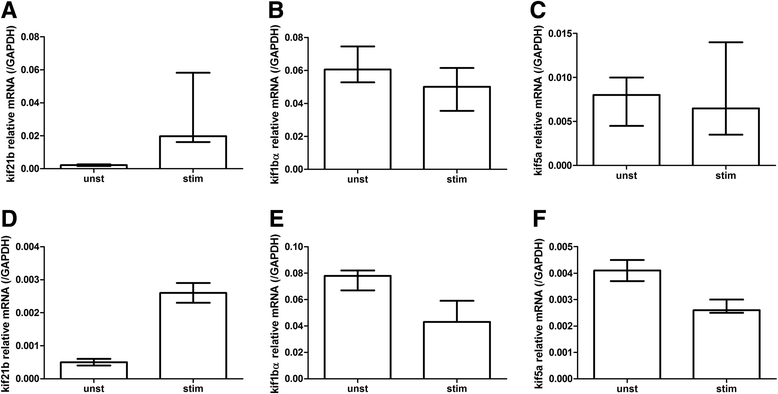

Supplement: Supplementary file 8 — Authors’ original file for figure 7 [file 40478_2014_9144_MOESM8_ESM.gif]

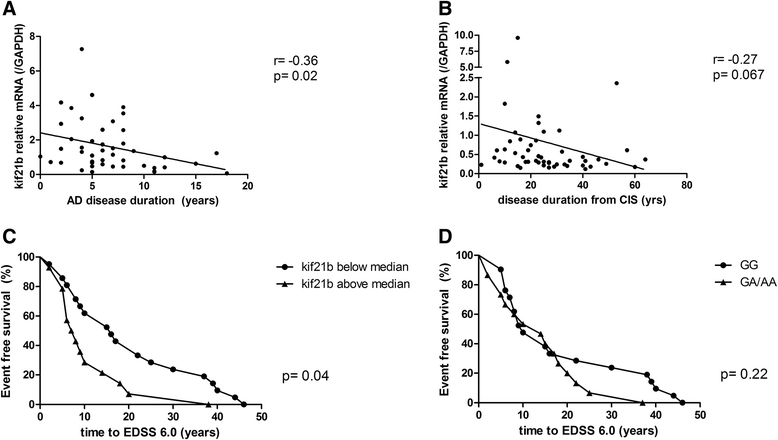

Supplement: Supplementary file 9 — Authors’ original file for figure 8 [file 40478_2014_9144_MOESM9_ESM.gif]
